# Distinct molecular patterns of TDP-43 pathology in Alzheimer’s disease: relationship with clinical phenotypes

**DOI:** 10.1186/s40478-020-00934-5

**Published:** 2020-04-29

**Authors:** Sandra O. Tomé, Rik Vandenberghe, Simona Ospitalieri, Evelien Van Schoor, Thomas Tousseyn, Markus Otto, Christine A. F. von Arnim, Dietmar Rudolf Thal

**Affiliations:** 1 Department of Imaging and Pathology - Laboratory of Neuropathology, and Leuven Brain Institute, KU-Leuven, O&N IV, Herestraat 49, box 1032, 3000 Leuven, Belgium; 2 Department of Neurosciences – Laboratory of Cognitive Neurology, KU- Leuven, Leuven, Belgium; 3 Department of Neurology, UZ Leuven, Leuven, Belgium; 4 Department of Neurosciences – Laboratory for Neurobiology, KU-Leuven and Center for Brain & Disease Research, VIB, Leuven, Belgium; 5 Department of Imaging and Pathology - Translational Cell and Tissue Research Unit, KU-Leuven, Leuven, Belgium; 6 Department of Pathology, UZ Leuven, Leuven, Belgium; 7 Department of Neurology, Ulm University, Ulm, Germany; 8 Department of Geriatrics, Göttingen University, Göttingen, Germany

**Keywords:** Alzheimer’s disease (AD), Frontotemporal lobar degeneration (FTLD), TDP-43, Protein aggregation, Phosphorylation

## Abstract

The co-existence of multiple pathologies and proteins is a common feature in the brains of cognitively impaired elderly individuals. Transactive response DNA-binding protein (TDP-43) has been discovered to accumulate in limbic brain regions of a portion of late-onset Alzheimer’s disease (AD) patients, in addition to amyloid-β and τ protein. However, it is not yet known whether the TDP-43 species in the AD brain differ in their composition, when compared among different AD cases and to frontotemporal lobar degeneration cases with TDP-43 inclusions (FTLD-TDP). Furthermore, it is not known whether TDP-43 pathology in AD is related to symptoms of the frontotemporal dementia (FTD) spectrum. In this study, we investigated the molecular pattern of TDP-43 lesions with five different antibodies against different phosphorylated (pTDP-43) and non-phosphorylated TDP-43 epitopes. We analyzed a cohort of 97 autopsy cases, including brains from 20 non-demented individuals, 16 cognitively normal pathologically-defined preclinical AD (p-preAD), 51 neuropathologically-confirmed AD cases and 10 FTLD-TDP cases as positive controls. We observed distinct neuropathological patterns of TDP-43 among AD cases. In 11 neuropathologically-confirmed AD cases we found dystrophic neurites (DNs), neuronal cytoplasmic inclusions (NCIs) and/or neurofibrillary tangle (NFT)-like lesions not only positive for pTDP-43^409/410^, but also for pTDP-43 phosphorylated at serines 403/404 (pTDP-43^403/404^) and non-phosphorylated, full-length TDP-43, as seen with antibodies against C-terminal TDP-43 and N-terminal TDP-43. These cases were referred to as AD^TDP + FL^ because full-length TDP-43 was presumably present in the aggregates. FTLD-TDP cases showed a similar molecular TDP-43 pattern. A second pattern, which was not seen in FTLD-TDP, was observed in most of p-preAD, as well as 30 neuropathologically-confirmed AD cases, which mainly exhibited NFTs and NCIs stained with antibodies against TDP-43 phosphorylated at serines 409/410 (pTDP-43^409^, pTDP-43^409/410^). Because only phosphorylated C-terminal species of TDP-43 could be detected in the lesions we designated these AD cases as AD^TDP + CTF^. Ten AD cases did not contain any TDP-43 pathology and were referred to as AD^TDP-^. The different TDP-43 patterns were associated with clinically typical AD symptoms in 80% of AD^TDP + CTF^ cases, 63,6% of AD^TDP + FL^ and 100% of the AD^TDP-^ cases. On the other hand, clinical symptoms characteristic for FTD were observed in 36,4% of AD^TDP + FL^, in 16,6% of the AD^TDP + CTF^, and in none of the AD^TDP-^ cases. Our findings provide evidence that TDP-43 aggregates occurring in AD cases vary in their composition, suggesting the distinction of different molecular patterns of TDP-43 pathology ranging from AD^TDP-^ to AD^TDP + CTF^ and AD^TDP + FL^ with possible impact on their clinical picture, i.e. a higher chance for FTD-like symptoms in AD^TDP + FL^ cases.

## Introduction

Alzheimer’s Disease (AD) is a progressive, neurodegenerative disorder and constitutes the most common form of dementia in people over 65 years of age [41]. AD is characterized by two main neuropathological hallmarks: extracellular amyloid-β (Aβ) deposition in senile plaques and intracellular generation of neurofibrillary tangles (NFTs), neuropil threads, and neuritic plaques containing abnormally phosphorylated τ protein (p- τ) [7]. Transactive response DNA-binding protein (TDP-43)-positive cytoplasmic inclusions in limbic areas occur in AD brains as well. They are present in up to 74% of AD cases [1, 2, 33, 40].

TDP-43 pathology has been described to expand in the AD brain in a stereotypical manner, starting in the amygdala and progressing into the medial temporal lobe and later into further regions such as temporal and frontal cortices [27, 47]. Furthermore, TDP-43 pathology in AD has been associated with a later onset of the disease, memory loss and hippocampal atrophy, playing a role in the clinical manifestation of the disease [28, 29]. Similar lesions have also been described in non-AD cases [40, 62].

Frontotemporal lobar degeneration with TDP-43 inclusions (FTLD-TDP) is a neurodegenerative disorder typically manifesting with behavioral changes or signs of aphasia [59], i.e., symptoms of the frontotemporal dementia (FTD) spectrum. Brettschneider et al. suggested that TDP-43 pathology spreads in a stereotypical manner in the behavioral variant of FTLD-TDP, with the prefrontal neocortex, middle-frontal gyrus, superior and middle-temporal gyri being heavily involved and with the amygdala being involved early in the disease [9]. FTLD-TDP has been classified into different subtypes, according to the morphology and topographic distribution of TDP-43 lesions in the cortex [39].

TDP-43-positive inclusions in limbic brain regions have been recently considered as limbic-predominant age-associated TDP-43 encephalopathy-related neuropathological changes (LATE-NC) [47]. This means that late-onset AD cases that have TDP-43 pathology may present concomitant LATE-NC, even without the clinical manifestation of LATE, according to the recent consensus working group report [47]. LATE-NC has recently been established as TDP-43 neuropathology in individuals without amyotrophic lateral sclerosis (ALS) or FTLD, which can be found in elderly individuals (mostly 80 years of age or older) with and without AD [47].

A controversial point raised by recent studies is whether TDP-43 deposition in AD cases represents the co-existence of AD and FTLD-TDP, or whether TDP-43 proteinopathy in AD is substantially different from that of FTLD-TDP [25–27, 49]. Given the recently developed, controversial concept of LATE-NC encompassing TDP-43 lesions in AD, non-AD and non-demented patients, the question whether TDP-43 pathology in these cases displays distinct molecular signatures is even more relevant. Furthermore, it was not yet addressed whether such patterns are different from that observed in FTLD-TDP [25].

TDP-43 is a nuclear protein that under pathological conditions can be cleaved and phosphorylated [10]. Due to the loss of the nuclear localization signal after cleavage, N-terminal truncated fragments of TDP-43 mislocalize and aggregate in the cytoplasm. In turn, the nucleus is depleted from normal TDP-43. Hence, a gain of toxic function in the cytoplasm as well as a loss of nuclear function seem to constitute TDP-43 disease mechanisms [50, 51]. Phosphorylation at serines 403/404 and 409/410 of TDP-43 constitutes pathological features of ALS and FTLD-TDP [2, 5, 18, 23, 48, 50]. However, the pattern of TDP-43 phosphorylation and species distribution present in AD cases is not yet understood.

Hence, we studied 97 autopsy cases including 20 non-AD/non-FTLD-TDP cases as controls, 16 pre-clinical AD cases, 51 neuropathologically-confirmed AD cases and 10 FTLD-TDP cases (used as positive controls). We screened our cohort with five different antibodies against several phosphorylated and non-phosphorylated TDP-43 epitopes. We report distinct molecular patterns of TDP-43 pathology among the cases with AD neuropathology based upon the detection of full-length TDP-43 or phosphorylated TDP-43 C-terminal fragments. These molecular differences were associated with a clinical presentation of AD or FTD symptoms.

## Material and methods

### Neuropathology

A total of 97 autopsy cases between 36 and 98 years of age (mean age: 72 years old, 45 females and 52 males) were investigated: 20 non-diseased controls, 16 pre-clinical AD, 51 neuropathologically-confirmed AD cases and 10 FTLD-TDP cases as positive controls for TDP-43 pathology (Table 1, Additional file 1-Table A1). Cases with hippocampal sclerosis were not included, since this pathology is considered as separate entity or separate type of LATE [1, 47, 54]. All autopsy brains were received from university or municipal hospitals in Leuven (Belgium), Bonn, Offenbach am Main and Ulm (Germany), and collected in accordance with local ethical committee guidelines and the federal laws governing the use of human tissue for research in Belgium and Germany. Dementia was diagnosed according to the DSM-IV criteria. The neuropathological diagnosis of AD was made when dementia was observed and when at least an intermediate degree of AD-related neuropathology was determined according to current criteria for the neuropathological diagnosis of AD as published by the National Institute of Aging and Alzheimer Association working group (NIA-AA criteria) [21]. The degree of dementia at the time of death was determined retrospectively using the Clinical Dementia Rating (CDR) score [19, 43]. For this purpose, the information from the clinical files was used to provide a CDR score. The CDR score was applied in controls and AD cases with AD clinical symptoms [43], whereas CDR with FTLD modules was used when scoring cases with a clinical picture of FTD [34]. The diagnosis of FTD behavioral variant and of primary progressive aphasia variants FTLD was made using consensus criteria [16, 53].

**Table 1:** Summary of the neuropathological groups used in this study. Mean age, Braak NFT stage, AβMTL phase, CERAD score and NIA-AA degrees of AD pathology are indicated. Number of cases with each LATE-NC stage and respective percentages are also indicated (see also Additional file 1-Table A1). n.a. not applicable

Neuropathological Diagnosis	Mean age	Mean Braak NFT-stage	Mean CERAD score	Mean NIA-AA degree	Mean AβMTL phase	No LATE-NC	LATE-NC stage 1	LATE-NC stage 2	LATE-NC stage 3	n
**Non-AD**	61	0,8	0	0	0	17 (85%)	1 (5%)	2 (10%)	0 (0%)	20
**p-preAD**	76	1,9	0,19	1	2	5 (31,2%)	3 (18,8%)	8 (50%)	0 (0%)	16
**AD**^**TDP-**^	78	3,7	1,4	2	3,5	10 (100%)	0 (0%)	0 (0%)	0 (0%)	10
**AD**^**TDP + CTF**^	76	5,1	2,4	2,7	3,9	1 (3,3%)	1 (3,3%)	19 (63,4%)	9 (30%)	30
**AD**^**TDP + FL**^	77	4,8	2,3	2,6	3,7	0 (0%)	0 (0%)	5 (45,5%)	6 (54,5%)	11
**FTLD-TDP**	65	1,2	0	0,4	0,6	n.a.	n.a.	n.a.	n.a.	10

The left hemispheres were fixed in formalin for 2 to 4 weeks and dissected. Blocks from frontal, parietal, temporal, occipital, and entorhinal cortex, the hippocampal formation at the level of the lateral geniculate body, basal ganglia, hypothalamus, thalamus, amygdala, basal nucleus of Meynert (NBM), midbrain, pons, medulla oblongata and cerebellum were embedded in paraffin. Five μm sections were cut using a microtome. The sections were stained with hematoxylin and eosin (H&E) for identification of pathologies different from AD and FTLD-related lesions.

### Immunohistochemistry

**Fig. 1:**
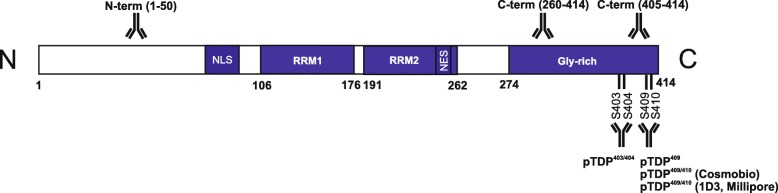
TDP-43 protein and epitopes targeted by the antibodies used in this study. N-t**-**TDP-43 (1-50aa., polyclonal, AVIVA systems), C-t-TDP-43 (260-414aa., polyclonal, ProteinTech), C-t-TDP-43 (405–410, polyclonal, Cosmobio), pTDP-43^403/404^ (polyclonal, Cosmobio), pTDP-43^409^ (polyclonal, Cosmobio), pTDP-43^409/410^ (polyclonal, Cosmobio) and pTDP-43^409/410^ (monoclonal, clone 1D3, Millipore)

Sections of the hippocampus, entorhinal, frontal, temporal and occipital cortex, amygdala, and NBM were stained with antibodies against TDP-43. Briefly, after epitope retrieval in heated citrate buffer (pH 6) and deparaffinization (using a Dako autostainer Link 48, Dako, Glostrup, Denmark), the sections were treated with peroxidase blocking reagent (Envision flex Peroxidase-Blocking Reagent, Dako) for 5 min. Primary antibodies (Additional file 1-Table A2) were applied for 30 min or overnight. The antibodies against TDP-43 provide the ability to detect specific sites of TDP-43 protein as well as the phosphorylation status at serines 403/404 and 409/410 (Fig. 1). Afterwards, the slides were incubated with an appropriate secondary antibody (Envision Dual flex, Dako). 3.3′-diaminobenzidine (Liquid DAB+ Substrate Chromogen System, Dako) was used as a chromogen to yield brown reaction products. Counterstaining with hematoxylin was performed. In sections of the hippocampus, entorhinal, temporal and occipital cortex, Aβ and p-τ immunostaining was performed using a similar protocol as described above, but with formic acid pre-treatment for antigen retrieval prior to the immunostaining procedure.

For immunofluorescence procedures, paraffin sections were incubated overnight with a cocktail of antibodies of different species (mouse TDP-43 C-terminus, rabbit TDP-43 N-terminus) (Additional file 1- Table A2) after the respective pre-treatment and deparaffinization. The sections were then incubated with a goat Cy2-labelled anti-mouse and goat Cy3-labelled anti-rabbit antibody cocktail (Jackson ImmunoResearch Ltd., West Grove, PA, USA) and mounted with ProLong™ Gold with DAPI (ThermoFisher Scientific, Rockford, IL, USA). The sections were microscopically analyzed with a Leica DM2000 LED microscope and images were taken with a Leica DFC7000 T camera, at 200 or 400x magnification.

The extent of p-τ, Aβ and TDP-43 pathology was assessed with antibodies against p-τ, Aβ and TDP-43 (Additional file 1-Table A2).

### TDP-43 pathology analysis

**Fig. 2:**
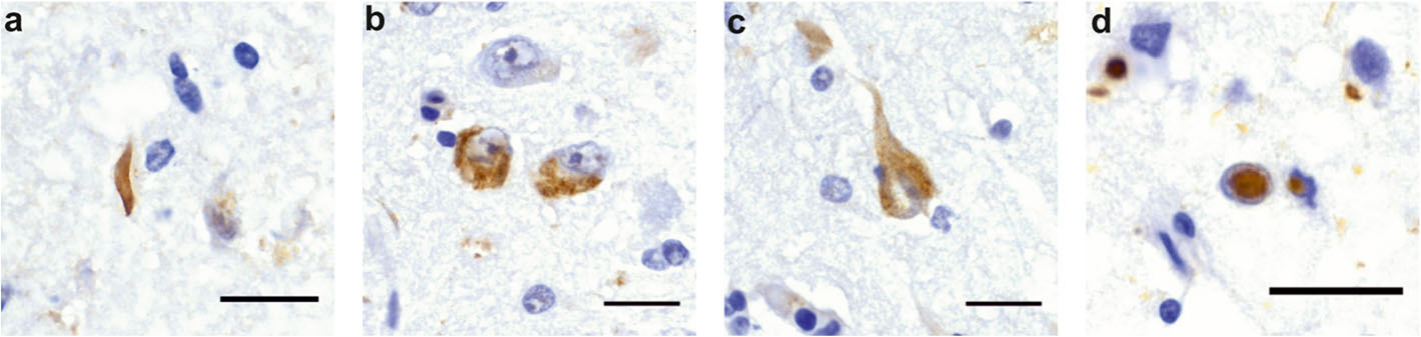
TDP-43 forms distinct types of lesions. Immunohistochemistry of an AD case with an anti-pTDP-43^409/410^ antibody (Cosmobio). **a** dystrophic neurite – DN, **b** neuronal cytoplasmic inclusions – NCI, **c** neurofibrillary tangle-like structure – NFT and (**d**) neuronal intranuclear inclusion – NII. Scale bar = 25 μm

A case was considered as TDP-43-positive in a given brain region, if there was TDP-43 immunoreactivity for one or more of the following lesions: neuronal cytoplasmic inclusions (NCIs), dystrophic neurites (DNs) or neuronal intranuclear inclusions (NIIs) (Fig. 2). Glial inclusions were not separately assessed, since none of our cases showed glial inclusions without associated neuronal TDP-43 pathology. Of note, a case was only considered to be TDP-43 negative when no TDP-43-positive lesion of any type, including glial inclusions, was detected. The identification of TDP-43 pathology was based on stainings with antibodies raised against pTDP-43^409/410^. pTDP-43^409/410^ staining in granulovacuolar degeneration (GVD) was not considered as a relevant TDP-43 lesion in this study, because it contains other phosphorylated proteins, such as compounds of the necrosome [35, 65] and it is not associated with nuclear clearance of TDP-43 [47]. NCIs, DNs and NIIs positive for TDP-43 have been previously associated with ALS and FTLD-TDP, as well as with AD [1, 18, 29, 48, 58, 66]. pTDP-43 pathology was analyzed for the relevant phosphorylation sites with antibodies against pTDP-43^409/410^, pTDP-43^409^ and pTDP-43^403/404^ (Additional file 1-Table A2). For confirmation, a monoclonal rat (clone 1D3) antibody against pTDP-43^409/410^ was used. The non-phosphorylated TDP-43 C-terminus (C-t TDP-43) was stained with a rabbit polyclonal antibody raised against the non-phosphorylated amino acids 260–414 of TDP-43 and confirmed with a rabbit polyclonal antibody against the non-phosphorylated amino acids 405–414 (Additional file 1-Table A2). The N–terminus of TDP-43 (N-t TDP-43) was detected with a rabbit polyclonal antibody raised against the amino acids 1–50 (Fig. 1).

TDP-43 pathology types α and β as defined by Josephs et al. were determined in non-FTLD-TDP/non-ALS cases, as proposed [26], by using sections stained with anti-pTDP-43^409/410^ antibodies. The presence of DNs and NCIs in the amygdala, hippocampal formation, and the frontotemporal cortex were classified as type α whereas cases with NFT-like pathology restricted to limbic regions (amygdala and hippocampus) were classified as type β. In the event that cases exhibiting a type α distribution pattern also presented NFT-like TDP-43 pathology, we considered them as type α + β (Additional file 1- Table A1). This classification was applied by two independent observers (ST and EVS) with an 85,3% agreement rate. Adjustment for discrepancies was reduced in a consensus diagnosis after discussing the results together at the microscope. AD cases with widespread lesions in the temporal or frontal cortex and FTLD-TDP cases were subtyped into the known FTLD-TDP subtypes (A-D), as defined by Mackenzie et al. [38], by analyzing the morphology and topography of DNs and NCIs in temporal and frontal cortices (see Additional file 1-Table A1). If a case did not fit into any of these subtypes, as it presented large NCIs, scattered among all layers of the temporal cortex, little DNs and occasionally NFT-like lesions, it was not further classified (indicated by * in Additional file 1-Table A1). AD cases without TDP-43 pathology in cortical layers were also not further classified into an FTLD-TDP subtype A-D, as this classification requires TDP-43 pathology in cortical layers. For the assessment of LATE-NC pathology, cases with more than 80 years of age and TDP-43 pathology in limbic regions (amygdala and hippocampal formation) without exclusive FTD-related symptoms were considered to be positive [47]. Cases younger than 80 years of age but older than 60 years with severe TDP-43 pathology restricted to limbic regions were also considered to represent LATE-NC. The amygdala, hippocampal formation and frontal cortex were used to stage for LATE-NC as proposed: Stage 0 = no LATE-NC; stage 1 = TDP-43 pathology restricted to the amygdala; stage 2 = TDP-43 pathology in the amygdala and hippocampal formation; stage 3 = TDP-43 pathology extending to the frontal cortex, in addition to stage 2 regions. LATE-NC staging was based on sections stained with a polyclonal antibody against pTDP-43 (pS409/pS410, Cosmobio). These criteria were not applied to the FTLD-TDP cases (positive controls). Of note, none of our cases fulfilled the criteria for hippocampal sclerosis according to the consensus criteria [54]. Genetic mutations (i.e. *C9ORF72*) in some cases are also referred in Additional file 1-Table A1.

### Aβ and τ pathology assessment

To determine the anatomical distribution of Aβ plaques, phases of Aβ deposition in the medial temporal lobe (AβMTL phases) were assessed as follows: Aβ plaques in the temporal neocortex (layers III, V and VI) characterize AβMTL phase 1. In AβMTL phase 2, the plaque deposition spreads into the layers pre-β – pri-γ of the entorhinal cortex, CA1, and the subiculum. AβMTL phase 3 is characterized by Aβ deposition in all six layers of the temporal neocortex including subpial band-like Aβ accumulation. In addition, Aβ plaques also occur in the outer molecular layer of the dentate gyrus and the parvopyramidal cell layer of the presubicular region. Finally, in AβMTL phase 4 there is fully developed β-amyloidosis in the medial temporal lobe with additional Aβ plaques in the CA4 region of the hippocampus and in the pre-α layer of the entorhinal cortex [64].

NFT distribution was assessed using the Braak NFT-staging method: Stage I is characterized by p-τ-positive neurons and threads, limited to the transentorhinal region, stage II by p-τ pathology in the entorhinal region, extending to CA1 and CA2, stage III by affection of the neocortex of the fusiform and lingual gyri, stage IV by progression into superior temporal neocortex areas and the dentate gyrus, stage V by the involvement of frontal and occipital cortex, reaching the peristriate region (layer V also begins to be affected) and finally, stage VI is identified by p-τ pathology in secondary and primary neocortical areas and extends into striate area of the occipital lobe [6, 7]. The consortium to establish a registry for AD (CERAD) scores for neuritic plaque density were assessed based on sections stained with an antibody against p-τ (AT8, Additional file 1-Tab. A1) [42]. The National Institute of Aging - Alzheimer Association (NIA-AA) degree of AD-pathology was determined according to Hyman et al. [21], based on the AβMTL phase, Braak NFT-stage, and the CERAD score for neuritic plaque pathology. CAA was diagnosed whenever Aβ deposits were found in the wall of cerebral and leptomeningeal blood vessels [14]. NFT pathology in the absence of Aβ plaque pathology was classified as definite primary age-related tauopathy (PART) [11].

### Statistical analysis

Non-parametric, Kruskal-Wallis H-test was used for independent samples, to perform comparisons between disease groups (Braak NFT-stage, AβMTL phase, NIA-AA, Age at death, CDR score). Friedman test for related samples was used to compare different antibodies within the same disease group. Bonferroni correction for multiple testing was applied. Post-hoc power analysis was calculated based on mean values with the help of G-power software (University of Düsseldorf, Germany).

Binary logistic regression was used to assess associations between the molecular TDP-43 patterns found in this study with clinical symptoms, AD-related neuropathological changes (Braak NFT-stages and AβMTL phases) and to associate these patterns with the morphological subtypes defined by Josephs et al. [26]. These analyses were controlled for age at death and sex, with a 95% confidence interval (CI).

Multinomial regression controlled for age and sex was used to further confirm the results obtained by Kruskal-Wallis H-test. For these regressions we used the symptomatic AD group as reference group.

IBM SPSS software (IBM, USA) was used in all instances.

## Results

Here, we analyzed the biological patterns of TDP-43 proteinopathy in demented cases with moderate-high degrees of AD pathology, pathologically defined pre-clinical AD (p-preAD) cases [61], FTLD-TDP cases and control cases (non-AD), by screening the whole cohort with five different TDP-43 antibodies. We distinguished distinct molecular patterns of TDP-43 pathology based on the different TDP-43 species and on the pattern of TDP-43 phosphorylation sites. Ten AD cases showed no TDP-43 pathology and were referred to as AD^TDP-^ cases. Thirty AD cases were positive for NCIs and NFT-like lesions stained with antibodies raised against pTDP-43^409/410^, as well as pTDP-43^409^, but neither with anti-pTDP-43^403/404^ nor with antibodies against C- or N-terminus epitopes of TDP-43. This subgroup of AD cases was designated as AD^TDP + CTF^. Of note, there was one exceptional AD case with predominant pTDP-43^409/410^ epitope expression that also had very sparse N-t TDP-43 pathology in amygdala and temporal cortex, but no pTDP^403/404^-positive lesions. For this reason, we classified this case also as an AD^TDP+CTF^ case. The remaining 11 neuropathologically-confirmed AD cases were positive not only for anti-pTDP43^409/410^ or anti-pTDP43^409^, but also for anti-pTDP-43^403/404^ and antibodies raised against non-phosphorylated TDP-43 species, such as C-t TDP-43 and N-t TDP-43. FTLD-TDP cases showed a similar expression pattern of these TDP-43 epitopes. Given the similarity of the TDP-43 staining pattern with the predominant expression of full-length TDP-43, the AD cases exhibiting all types of TDP-43 species were referred to as AD^TDP + FL^. Furthermore, 7 out of these 11 AD^TDP + FL^ cases presented a morphological pattern of TDP-43 pathology compatible with one of the FTLD-TDP subtypes A-C as described before [39], whereas the remaining four cases did not fit into any of these subtypes (Additional file 1-Table A1).

Clinically, most AD^TDP + CTF^ cases exhibited a typical AD phenotype during life (80%) and 13,3% presented primary progressive aphasia (PPA), whereas the AD^TDP + FL^ cases showed variable clinical phenotypes with AD (63,6%) and/or FTD-related symptoms (36,4%), such as behavioral problems and personality changes (Table 2).

**Table 2:** Summary of the neuropathological groups used in this study. The number of cases with each observed symptom(s) and respective percentages are indicated (see also Additional file 1-Table A1)

Neuropathological Diagnosis	Non-demented	Typical AD	FTD symptoms with or without additional AD symptoms						n
			svPPA	svPPA + AD	AD + behavior	AD + motor speech	bvFTD	PSP	
**Non-AD**	20 (100%)	0 (0%)	0 (0%)	0 (0%)	0 (0%)	0 (0%)	0 (0%)	0 (0%)	20
**p-preAD**	16 (100%)	0 (0%)	0 (0%)	0 (0%)	0 (0%)	0 (0%)	0 (0%)	0 (0%)	16
**AD**^**TDP-**^	0 (0%)	10 (100%)	0 (0%)	0 (0%)	0 (0%)	0 (0%)	0 (0%)	0 (0%)	10
**AD**^**TDP + CTF**^	0 (0%)	24 (80%)	4 (13,4%)	0 (0%)	1 (3,3%)	1 (3,3%)	0 (0%)	0 (0%)	30
**AD**^**TDP + FL**^	0 (0%)	7 (63,6%)	0 (0%)	1 (9,1%)	1 (9,1%)	0 (0%)	2 (18,2%)	0 (0%)	11
**FTLD-TDP**	0 (0%)	0 (0%)	3 (30%)	0 (0%)	1 (10%)	0 (0%)	5 (50%)	1 (10%)	10

### Distinct patterns of TDP-43 and its modified forms in cases fulfilling the neuropathological criteria for AD

To clarify the biological differences between TDP-43 patterns in AD cases, we investigated the prevalence of pTDP-43 and non-phosphorylated TDP-43 species. For that, we used phospho-dependent and phospho-independent antibodies against the N- and C-t epitopes of TDP-43 (Fig. 1). To test whether the TDP-43 staining patterns are consistent among different brain regions we analyzed hippocampal sub-regions (dentate gyrus, CA4, CA3/2, CA1, subiculum), amygdala, basal nucleus of Meynert (NBM), entorhinal, temporal, frontal and occipital cortices.

**Fig. 3:**
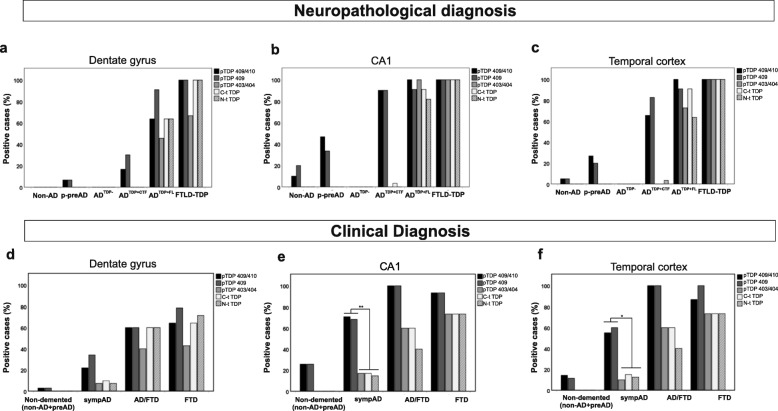
Amount of positive cases for lesions detected with pTDP-43^409/410^, pTDP-43^409^, pTDP-43^403/404^, C- and N-t-TDP-43 in (**a**, **d**) dentate gyrus, (**b**, **e**) CA1 and (**c**, **f**) temporal cortex, according to a neuropathological classification (non-AD, p-preAD, AD^TDP-^, AD^TDP + CTF^, AD^TDP + FL^ and FTLD-TDP) (**a-c**), or further grouped by clinical diagnosis (non-demented – including neuropathological non-AD and p-preAD cases, sympAD – all cases with an exclusive AD clinical presentation, AD/FTD – cases with signs of AD as well as FTD symptoms, and FTD – cases with an exclusive FTD clinical presentation) (**d-f**). Non-parametric, Friedman test for related samples with Bonferroni correction for multiple testing was used to compare the amount of positive cases with each TDP-43 antibody within each clinical group. Each group was analyzed separately, *p < 0.05, **p < 0.01

**Fig. 4:**
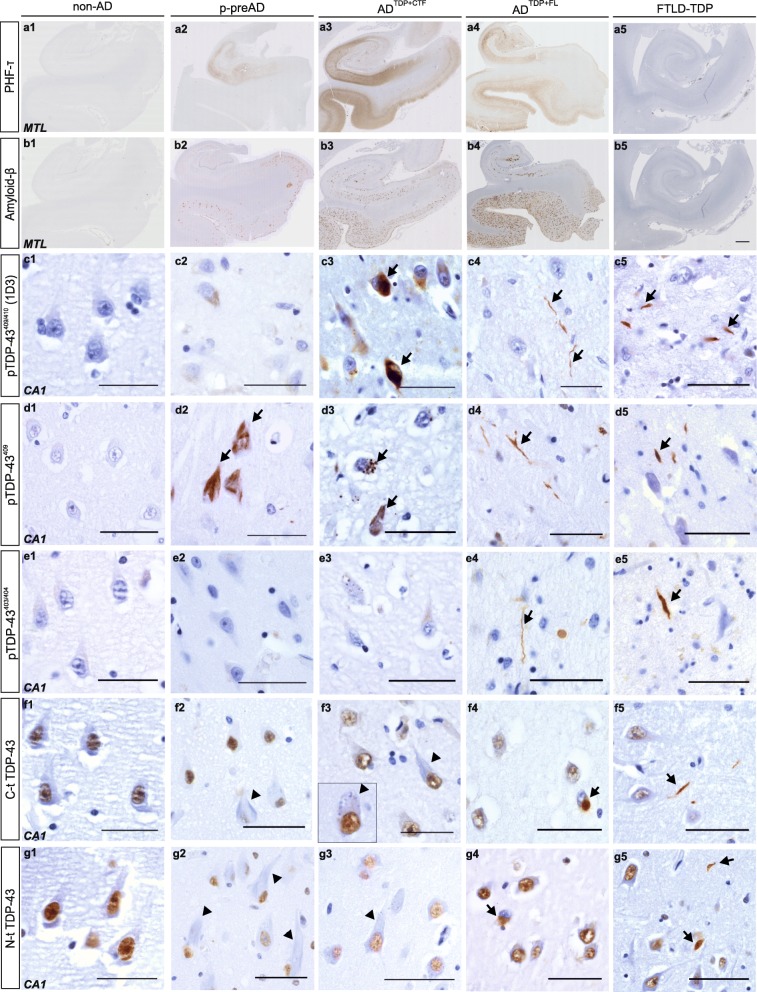
pTDP-43 species are predominant in AD^TDP + CTF^ whereas full-length TDP-43 is abundant in AD^TDP + FL^ and FTLD-TDP**.** Immunohistochemistry of a non-AD, p-preAD, AD^TDP + CTF^, AD^TDP + FL^ and FTLD-TDP case in the medial temporal lobe (**a1-b5**) with (**a**) PHF-τ, (**b**) Aβ (4G8). Scale bar = 1 mm. CA1-hippocampus (**c1-g5**) of a non-AD, p-preAD, AD^TDP + CTF^, AD^TDP + FL^ and FTLD-TDP with (**c**) pTDP-43^409/410^ (clone 1D3), (**d**) pTDP-43^409^, (**e**) pTDP-43^403/404^ (**f**) C-t TDP-43 and (**g**) N-t TDP-43 antibodies. NCIs and NFT-like lesions are detected in p-preAD and AD^TDP + CTF^ cases with pTDP-43^409/410^ (**c3**, arrows) and pTDP-43^409^ (**d2-d3**, arrows), but not pTDP-43^403/404^ (**e2-e3**). Nuclei are stained with C- and N-t-TDP-43 antibodies and unstained, “ghost” NFTs, NCIs and GVD are apparent (**f2,g2-g3** arrowheads, **f3** inset, arrowheads) in p-preAD and AD^TDP + CTF^ cases. NCIs and DNs are detected in AD^TDP + FL^ and FTLD-TDP with pTDP-43^409/410^ (**c4-c5** respectively, arrows), pTDP-43^409^ (**d4-d5** respectively, DNs, arrows), pTDP^403/404^ (**e4-e5** respectively, DNs, arrows), C-t (**f4-f5** respectively, NCI and DN are indicated with arrows, respectively) and N-t-TDP-43 (**g4-g5**, NCI and two DNs are indicated with arrows, respectively). AD^TDP-^ cases were not included in this figure because no TDP-43 inclusions were observed. Scale bar = 50 μm

The number of positive cases for pTDP^409^ and pTDP^409/410^ in most regions analyzed in this study were similar among AD^TDP + CTF^ and AD^TDP + FL^, with the exception of dentate gyrus, CA4, frontal and occipital cortex for pTDP^409/410^ and dentate gyrus, occipital cortex for pTDP^409^. This was due to the low abundance of TDP-43 pathology in AD^TDP + CTF^ cases in these regions (Fig. 3 a-c, 4 Additional file 1- Fig. A1-A3, Tab. A3, 4). In turn, the predominance of TDP-43^403/404^ pathology in AD^TDP + FL^ cases compared to AD^TDP + CTF^ cases was higher in all regions analyzed except NBM and occipital cortex, which exhibited low pTDP-43^403/404^ pathology in both groups (Fig. 4, Additional file 1-Fig. A1-A3, A5). Furthermore, AD^TDP + FL^ cases displayed a higher percentage of cases positive for non-phosphorylated TDP-43 in all regions except NBM compared to AD^TDP + CTF^ cases, as seen with antibodies raised against the C-t TDP-43 (Fig. 4 a-c, Additional file 1-Fig. A3, A6). This was also true for the N-t-TDP-43 in all regions but CA4, CA3/2 and NBM (Fig. 4 a-c, Additional file 1-Fig. A3, A7).

Importantly, the differences between AD^TDP + CTF^ and AD^TDP + FL^ cases were analogous to those observed between AD^TDP + CTF^ and FTLD-TDP cases (Fig. 3 a-c, Additional file 1-Fig. A1-A3). To indicate the power of our findings, we performed a post-hoc power analysis regarding the analysis of the five different TDP-43 markers in the CA1 region of the hippocampus. We obtained a power of 80–90% for the different antibodies to distinguish between the neuropathologically defined groups.

Furthermore, when using antibodies against N-t and C-t, normal nuclear TDP-43 was detected. Non-stained “ghost” NFT-like structures and GVD were occasionally observed with these antibodies in p-preAD and AD^TDP + CTF^ cases (Fig. 4 f2-f3, g2-g3, arrowheads).

AD^TDP + FL^ and FTLD-TDP groups displayed a very similar neuropathological profile regarding all TDP-43 species in most regions (Fig. 3 a-c; Additional file 1-Fig. A1-A3). FTLD-TDP cases still displayed a higher abundance of pTDP^403/404^, C- and N-t TDP-43 lesions in most regions when compared to AD^TDP + FL^ cases (Additional file 1-Fig. A3).

Morphologically, a few non-AD, p-preAD and AD^TDP + CTF^ cases displayed GVD, large NCIs, few DNs and NFT-like material (Fig. 4 d2, c3-d3, arrows; Additional file 1-Fig. A1-e2-e3). AD^TDP + FL^ cases, as well as FTLD-TDP cases displayed abundant DNs (Fig. 4 c4–5, d4–5, e4–5; Additional file 1- Fig. A1-A2) as well as NCIs (Fig. 4 f4,g4, arrows; Additional file 1-Fig. A1-A2).

Of note, whenever inclusions were detected in the cytoplasm with antibodies against C- or N-t-TDP-43, the nucleus was depleted of normal TDP-43, namely in AD^TDP + FL^ and FTLD-TDP cases (Fig. 4 f4, g4; Additional file 1-Fig. A2-a4-a5 and d4-d5 arrows).

In addition, nuclear clearance of C-t TDP-43 was present in AD^TDP + CTF^ cases, and even more pronounced in AD^TDP + FL^ and FTLD-TDP cases. However, this was purely observational and it was not quantified.

**Fig. 5:**
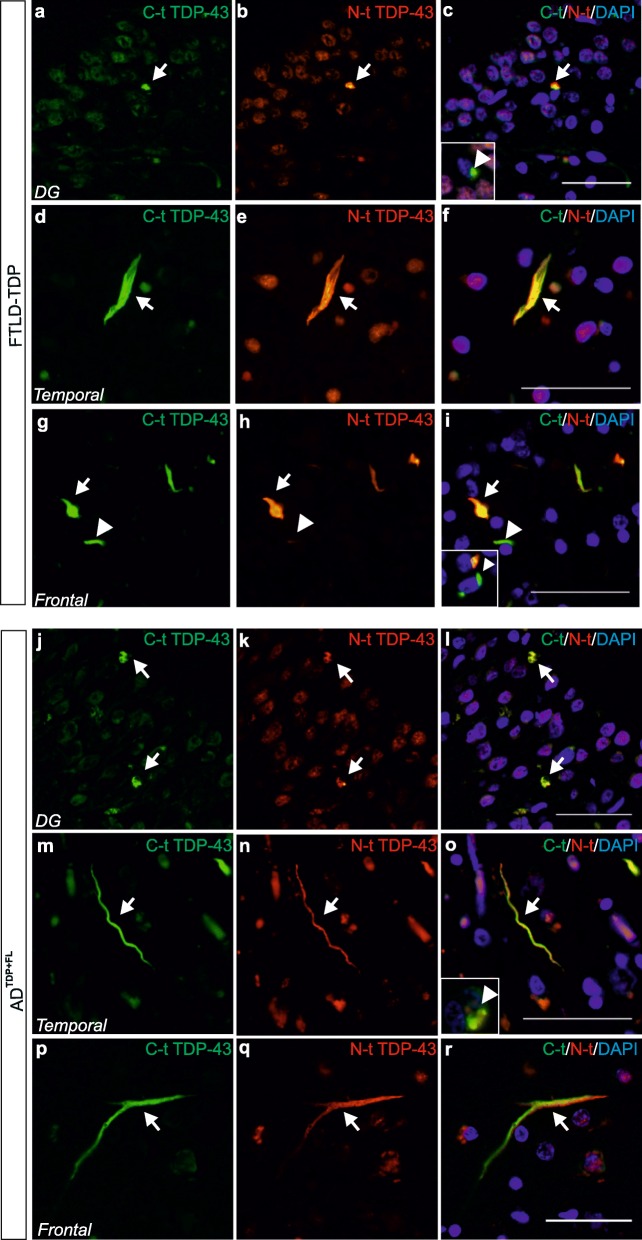
TDP-43 aggregates in AD^TDP + FL^ and FTLD-TDP mostly comprise the full-length protein or just CTFs. Double-labeling with N-t TDP-43 and C-t TDP-43 antibodies in a (**a**-**i**) FTLD-TDP, and (**j**-**r**) AD^TDP + FL^. The majority of NCIs and DNs positive for the full-length protein are detected in FTLD-TDP in the DG, temporal and frontal cortices (**c, f, i**, respectively, arrows) and in AD^TDP + FL^ cases in the DG, temporal and frontal cortices (**l, o, r**, respectively, arrows). C-t exclusive inclusions were detected in FTLD-TDP cases in the DG (**c, inset**, arrowhead) and in the frontal cortex (**g, h, i**, arrowheads and **i, inset**, arrowhead). C-t exclusive inclusions were also detected in AD^TDP + FL^ cases in the temporal cortex (**o, inset**, arrowhead). Scale bars = 50 μm

To further investigate the TDP-43 aggregate composition, we performed fluorescence double-labelling experiments with antibodies raised against N-t and C-t-TDP-43. We confirmed that the inclusions in FTLD-TDP as well as in AD^TDP + FL^ cases prominently exhibited the full-length protein at least in the dentate gyrus (Fig. 5 a-c, j-l), temporal cortex (Fig. 5 d-f,m-o) and frontal cortex (Fig. 5 g-i,p-r). A minority of inclusions were exclusively positive for C-t-TDP-43 in these regions as well (Fig. 5 c,i,o - insets).

### Distribution of TDP-43 pathology in AD and FTLD

In AD^TDP + FL^, as well as in FTLD-TDP cases, all regions were affected by pTDP-43^409/410^ and pTDP-43^409^ pathology in 50–100% of positive cases (Additional file 1-Tables A3-A4). This was also true for C-t TDP-43, where 45,5–100% cases of these two groups were positive for this antibody in the investigated regions except for NBM (Additional file 1-Table A6). Furthermore, all regions except for NBM and occipital cortex were positive for pTDP^403/404^ in 45.5–100% of cases AD^TDP + FL^ cases, whereas 50–100% of FTLD-TDP cases were positive for this antibody in the investigated regions, including NBM and occipital cortex (Additional file 1-Table A5). The number of severely affected regions was lower with an antibody against N-t-TDP-43. Nevertheless, in AD^TDP + FL^ cases at least seven regions were heavily impacted by N-t-TDP-43 pathology, and all investigated regions except for NBM showed lesions detectable with this antibody (Additional file 1-Table A7). Similarly, in FTLD-TDP cases, all regions except for NBM and occipital cortex were considerably affected by N-t TDP-43 positive lesions (Additional file 1-Table A7). This means that despite AD^TDP + FL^ and FTLD-TDP cases were molecularly similar in terms of TDP-43 species, FTLD-TDP cases showed a more widespread distribution of TDP-43 pathology.

On the other hand, in p-preAD cases, as well as AD^TDP + CTF^ cases, the pTDP-43^409/410^ and pTDP-43^409^ pathology seemed to be mainly restricted to the medial temporal lobe incl. Amygdala, with the exception of the dentate gyrus of the hippocampus, which was generally spared (Additional file 1-Tables A3). Therefore, the most affected regions were amygdala, CA1, subiculum and entorhinal cortex (56.7–90% of AD^TDP + CTF^ cases) whereas the frontal and occipital cortices were less affected (up to 44.8 and 14.3% of positive cases, respectively). Similarly, in the non-AD cases with pTDP-43^409/410^ pathology, these lesions were also restricted to the medial temporal lobe, incl. Amygdala.

### LATE-NC distribution in AD^TDP + CTF^ and AD^TDP + FL^

**Fig. 6:**
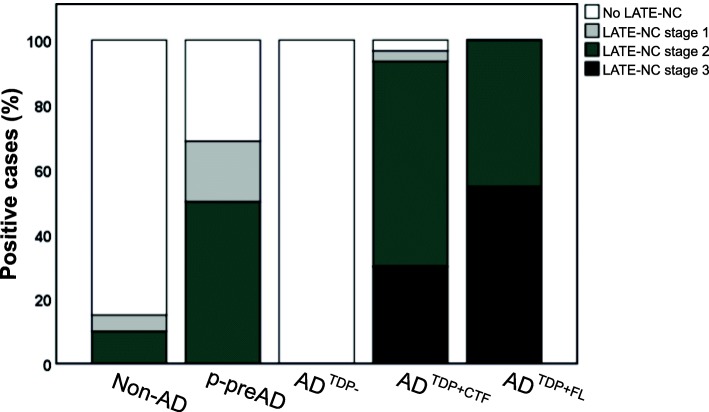
LATE-NC in non-AD, p-preAD, AD^TDP-^, AD^TDP + CTF^ and AD^TDP + FL^ cases. Most non-AD cases have no LATE-NC. AD^TDP-^ have no LATE-NC. The majority of p-preAD and AD^TDP + CTF^ cases present LATE-NC restricted to the medial temporal lobe (stage 2). One AD^TDP + CTF^ case was not considered for the LATE-NC classification due to being considerably younger, despite presenting TDP-43 pathology. On the other hand, nearly half of AD^TDP + FL^ cases have LATE-NC stage 2 and the remaining cases present more widespread LATE-NC, extended to the frontal cortex (stage 3)

We classified our cases that were not typical FTLD-TDP with the recently proposed staging scheme for TDP-43 pathology by Nelson and colleagues [47]. Therefore, we considered LATE-NC positive whenever cases were older than 60 years and had at least pTDP-43 pathology (Table 2, Additional file 1- Table A1). We observed that a minority of non-AD cases displayed LATE-NC stage 1 or 2 (5 or 10%, respectively), meaning that the TDP-43 pathology was limited to the amygdala and hippocampal formation, whereas the remaining control cases did not display LATE-NC (Fig. 6). This was either because they were too young to be considered part of the LATE-NC spectrum and/or because there was no TDP-43 pathology. Around half of p-preAD and AD^TDP + CTF^ cases showed TDP-43 pathology in LATE-NC stage 2, extending into the hippocampus (50% and 63,3%, respectively), whereas 31,3% of p-pre AD cases did not show LATE-NC. Additionally, 30% of AD^TDP + CTF^ cases displayed TDP-43 pathology extending to the frontal cortex (LATE-NC stage 3, Fig. 6).

As for the AD^TDP + FL^ cases, more than half of the cases (54,5%) presented LATE-NC stage 3, whereas the remaining cases were classified as stage 2 LATE-NC (Fig. 6). These findings corroborate our previous results and further demonstrate that in AD^TDP + CTF^ cases, TDP-43 pathology was mostly restricted to the limbic system, whereas the TDP-43 pathology in AD^TDP + FL^ cases was more frequently widespread in the brain.

### Relationship of molecular TDP-43 patterns with other AD-related neuropathological lesions

**Fig. 7:**
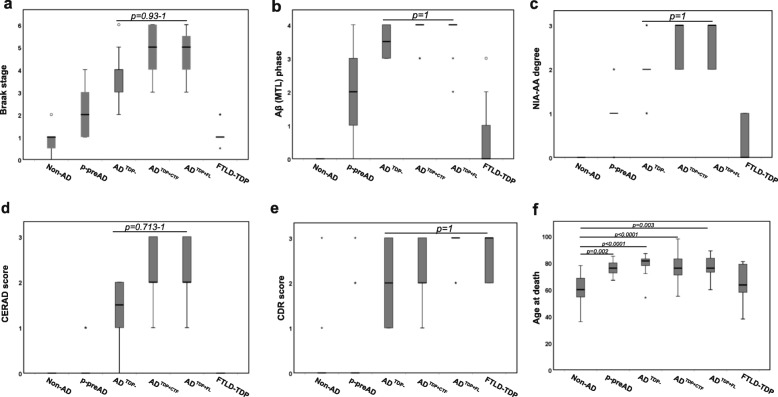
AD^TDP + FL^ cases have high amounts of p-τ, Aβ, and a high dementia score, comparable to AD^TDP + CTF^ cases. **a** Braak NFT-stage, **b** AβMTL phase, **c** NIA-AA degree of AD pathology, **d** CERAD score, **e** CDR score and (**f**) age at death among neuropathological groups. No significant differences were found between these AD groups except for age. p-preAD, AD^TDP-^, AD^TDP + CTF^ and AD^TDP + FL^ cases were significantly older when compared to non-AD controls (*p* = 0.002, *p* < 0.0001, p < 0.0001 and *p* = 0.003, respectively). Non-parametric, Kruskal-Wallis test for independent samples with Bonferroni correction for multiple testing was used to compare the same independent variable among neuropathological groups. When limiting the sample to the AD groups and comparing AD^TDP-^, AD^TDP + CTF^, and AD^TDP + FL^ cases, two by two with binary logistic regression analysis controlled for age and sex and adjusted for multiple testing by the Bonferroni procedure, AD^TDP + CTF^ cases showed higher Braak NFT stages, AβMTL phases, CERAD scores, and NIA-AA degrees of AD pathology compared to AD^TDP-^ cases (Additional file 1-Tables A8-A11)

Notably, AD^TDP + FL^ cases were not distinguishable from AD^TDP + CTF^ cases and AD^TDP-^ regarding Aβ, neuritic plaque and NFT pathology (Braak NFT stages III-VI, AβMTL phases 3–5; CERAD score: 1–3; NIA-AA stage 2–3) (*p* = 1, Fig. 4 a3-a4,b3-b4, Fig. 7 a-d). Kruskall-Wallis with Bonferroni correction for multiple testing was used. For these neuropathological parameters, a post-hoc power analysis revealed a power of 100% for the differences observed among all six groups of cases. The majority of non-AD, control cases (75%) presented PART (Fig. 7 a, Additional file 1-Table A1). Moreover, 2 out of the 3 non-AD cases with TDP-43 pathology mentioned earlier were contemplated among the PART group.

Furthermore, the dementia scores from AD^TDP + FL^ and FTLD-TDP cases (CDR score 2–3) were not significantly different from AD^TDP-^ as well as AD^TDP + CTF^ cases (CDR score 0.5–3) (p = 1, Fig. 7 e). By definition, no signs of dementia were observed in control and p-preAD cases.

Age at death differed significantly between groups when using Kruskall-Wallis test, specifically between non-AD controls and p-preAD: the p-preAD group was on average older than the controls (*p* = 0.002). The non-AD cases were also significantly younger than the AD^TDP-^ (*p* < 0.0001), AD^TDP + CTF^ (p < 0.0001), and AD^TDP + FL^ cases (*p* = 0.003, Fig. 7 f). There were no significant differences in the age of AD^TDP-^, AD^TDP + CTF^, AD^TDP + FL^ and FTLD-TDP cases (0.108 < *p* ≤ 1, Fig. 7f). In line with these results, we performed binary logistic regression to address the differences of Braak NFT-stages, AβMTL phases, CERAD scores and NIA-AA degrees of AD pathology among AD^TDP + CTF^ and AD^TDP + FL^ groups, when controlled for age and sex. We observed that there were no significant differences among these two groups and AD^TDP-^ with AD^TDP + FL^ cases in all parameters analyzed after correction for multiple testing (Additional file 1- Tables A8-A11). Only when comparing AD^TDP-^ with AD^TDP + CTF^ cases, higher Braak NFT stages, AβMTL phases, CERAD scores, and NIA-AA degrees of AD pathology were observed in AD^TDP + CTF^ cases (Additional file 1- Tables A8-A11).

We then tested by binary logistic regression (while controlling for age at death and sex) if there was an association between the molecular patterns identified here with the morphological types of TDP-43 lesions as previously described by Josephs et al. [26]. In this study, the authors defined two morphological types of TDP-43 pathology in non-FTLD brains: type α – DNs, NIIs or NCIs widespread in the brain, and type β – NFT-associated material, restricted to the medial temporal lobe. We classified our cases according to this system (see Additional file 1-Table A1). We further classified all our cases with the molecular patterns found in this study: ‘TDP + CTF’ pattern, positive for pTDP^409^, and pTDP^409/410^; or ‘TDP + FL+’ pattern, positive for all TDP-43 markers. The ‘TDP + CTF’ pattern (present in some controls, most p-preAD and all AD^TDP + CTF^ cases) was strongly associated with type β (p < 0.0001, Additional file 1-Table A12) characterized by NFT-like lesions. The ‘TDP + FL’ pattern (observed in AD^TDP + FL^ and FTLD-TDP cases) did neither show an association with type α nor with type β (Additional file 1-Table A13). We analyzed each molecular pattern individually due to collinearity effects. In our cases, we did not observe pure type α pattern. This pattern was always associated with at least few NFT-like TDP-43 inclusions and, therefore, considered as type α + β.

### Association of the TDP-43 molecular patterns with clinical phenotypes

To further investigate these molecular patterns statistically, we re-grouped our cohort according to the clinical diagnosis: non-demented (including the neuropathological non-AD and p-preAD cases, *n* = 36), symptomatic AD (sympAD = all cases with a typical AD phenotype, *n* = 40), AD/FTD (cases with signs of both AD and FTD symptoms such as behavioral and/or language problems, *n* = 5) and FTD cases (cases with a clinical FTD phenotype, *n* = 15). We found that even when grouping our cases based on clinical phenotype, we observed significant differences in the prevalence of pTDP^409/410^, pTDP^409^, pTDP^403/404^, C- and N-t-TDP-43 reactivity, particularly in sympAD cases (Fig. 3 d-f, Additional file 1-Fig. A4) whereas no differences were observed in non-demented individuals (Additional file 1-Table A14, A15). Specifically, in sympAD we observed significant differences between the positivity for both pTDP^409/410^ and pTDP^409^ when compared to pTDP^403/404^, C- and N-t TDP43 in CA1 region and temporal cortex (*p* ≤ 0.001 and *p* < 0.047, Fig. 3 e-f). Moreover, significant differences were observed in the staining pattern between these antibodies in the remaining sub-regions of the hippocampus such as CA4, CA3/2, subiculum, as well as entorhinal, amygdala and NBM in sympAD (*p* < 0.01, Additional file 1-Fig. A4a-f, Additional file 1-Table A15). SympAD cases displayed a lower prevalence of all antibodies in frontal and occipital cortices, therefore there were no significant differences in these regions (Additional file 1-Fig. A4g-h Additional file 1-Table A15).

Moreover, there were no significant differences in positivity of the TDP-43 lesions between the anti-TDP-43 antibodies in the clinical AD/FTD group nor in the FTD group (Additional file 1-Tables A16-A17).

To further confirm the differences in the molecular profile among the clinical groups, we used multinomial logistic regression controlled for age and sex in the most severely affected region (CA1). For this, we used the sympAD group as a reference category. Consistent with our previous results, we observed significant differences between the sympAD and the FTD group regarding the prevalence of pTDP^409/410^ (*p* = 0.023), pTDP-43^403/404^ (*p* = 0.001), C- and N-t TDP-43 (*p* = 0.001), but not pTDP-43^409^ (Additional file 1-Tables A18-A22). This strengthens the hypothesis that the patterns of TDP-43 species differ among these dementias. Furthermore, the positivity for pTDP-43^409/410^ and pTDP-43^409^ was lower in non-demented cases, when compared to sympAD cases (Additional file 1-Tables A18-A19). Of note, age at death of FTD cases was lower than that of the sympAD cases. This was also true for non-demented cases (Additional file 1-Tables A18-A22). When using age as the only independent variable through multinomial logistic regression, we confirmed that the sympAD cases were significantly older than the FTD cases (*p* = 0.002, odds ratio = 1.1).

Clinically, all AD^TDP-^ cases had a clinical AD phenotype (Table 2). Twenty-four out of 30 AD^TDP + CTF^ cases (80%) presented typical AD symptoms in life, such as amnestic deficits and executive dysfunction. Four AD^TDP + CTF^ cases (13,3%) exhibited prominent language problems, with an initial diagnosis of semantic variant-primary progressive aphasia (svPPA). Interestingly, one AD^TDP + CTF^ case (3,3%) showed an AD clinical phenotype and motor speech deficits later on in the disease. Another AD^TDP + CTF^ case (3,3%) presented both clinical AD and behavioral problems, such as impulsivity and aggression (Table 2).

The AD^TDP + FL^ cases presented a larger variety of symptoms ranging from AD to FTD features. Specifically, 7 out of 11 AD^TDP + FL^ cases (63,6%) exhibited a classical AD clinical phenotype – memory deficits and executive dysfunction - whereas 2 AD^TDP + FL^ cases (18,2%) had a diagnosis of the behavioral variant of FTD (bvFTD), with pronounced behavioral problems. Moreover, 1 AD^TDP + FL^ case (9,1%) with the *C9ORF72* mutation displayed behavioral FTD-like deficits as well as AD symptoms such as memory deficits. Finally, one AD^TDP + FL^ case (9,1%) displayed svPPA during life, with additional AD signs later on (Table 2, Additional file 1-Table A1).

As for FTLD-TDP cases, 5 out of 10 cases (50%) presented a bvFTD clinical presentation, 3 cases (30%) had svPPA, one case (10%) presented an AD phenotype but later evolved to a bvFTD-like presentation. Another FTLD-TDP case (10%) displayed clinical signs of progressive supranuclear palsy (PSP), due to additional PSP neuropathology (Table 2).

Interestingly, we observed that the AD^TDP + FL^ cases with a Josephs’ morphological pattern type β in the absence of type α features (see additional file 1- Table A1) were clinically typical AD whereas the presence of type α features was observed in 57.1% of the AD^TDP + FL^ cases with FTD symptomatology. To address this, we performed a binary logistic regression using Josephs’ type α as a dependent variable and FTD symptoms, age at death and sex as independent variables. We observed an association between Josephs’ type α and FTD symptoms (*p* = 0.039), but not between type β and FTD symptoms (*p* = 0.999), as expected (Additional File 1-Tables A23–24).

Finally, we addressed whether the different TDP-43 molecular patterns identified in this study are statistically associated with different clinical manifestation of the disease. For this, we only considered the demented cases with TDP-43 pathology and performed binary logistic regressions, while controlling for age at death and sex. We found that the ‘TDP + CTF’ molecular pattern was statistically associated with typical AD symptoms - amnestic syndrome executive dysfunction – and that age at death was also associated with clinical AD (Table 3) but inversely associated with FTD symptoms (Additional file 1- Table A25). In turn, the ‘TDP + FL’ pattern, observed in AD^TDP + FL^ as well as FTLD-TDP cases, was significantly associated with a clinical presentation of the FTD spectrum – cases that presented behavioral problems or language deficits (Table 4) - and inversely associated with clinical AD (Additional file 1- Table A26). Post-hoc power analysis of these comparisons among the clinical phenotypes revealed a power 48–87% when considering the typical AD symptoms (87%) and FTD symptoms with or without AD-type cognitive impairment (48%) in each neuropathological group.

**Table 3:** Binary logistic regression addressing the statistical association between typical AD symptoms and the ‘TDP + CTF’ pattern. Only demented cases with TDP-43 pathology, incl. FTLD-TDP, were included

Typical AD symptoms				
	Sig.	Odds ratio	95%CI for Odds ratio:lower	95%CI for Odds ratio:upper
**TDP + CTF Pattern**	0,003	7,916	1,999	31,314
**Age at death**	0,034	1,085	1,006	1,169
**Sex**	0,530	1,559	0,389	6,338

**Table 4:** Binary logistic regression addressing the statistical association between FTD symptoms and the ‘TDP + FL’ pattern. Only demented cases with TDP-43 pathology, incl. FTLD-TDP, were included

FTD symptoms				
	Sig.	Odds ratio	95%CI for Odds ratio:lower	95%CI for Odds ratio:upper
**TDP + FL Pattern**	0,022	5,633	1,285	24,684
**Age at death**	0,036	0,926	0,861	0,995
**Sex**	0,225	2,476	0,573	10,702

## Discussion

In our study, we aimed to clarify the molecular characteristics of TDP-43 aggregates in AD cases with TDP-43 pathology.

**Fig. 8:**
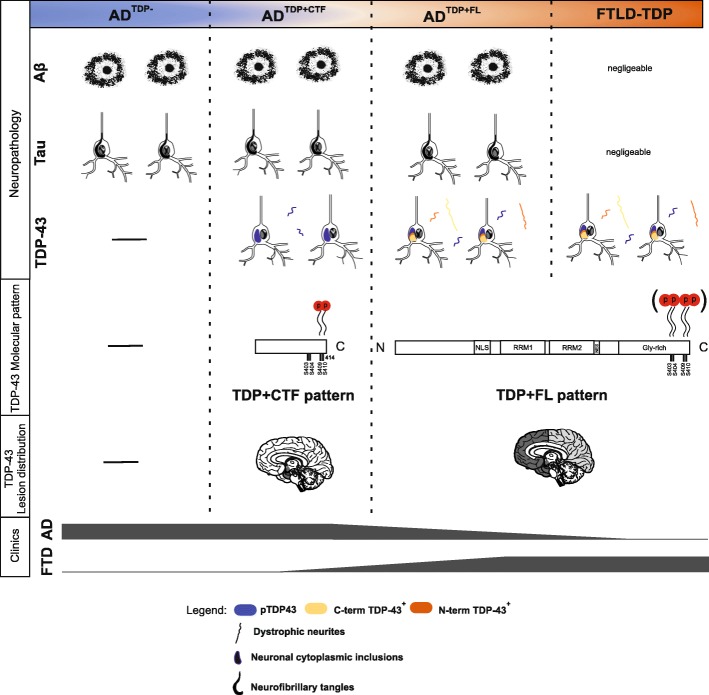
TDP-43 pathology presents distinct molecular patterns in AD^TDP + CTF^ vs. AD^TDP + FL^ and FTLD-TDP. TDP-43 lesions in AD cases consist of NCIs, NFT-like structures and few DNs, which are mostly positive for pTDP-43^409^ and pTDP-43^409/410^ presumably representing aggregates of N-terminal truncated pTDP-43^409/410^. Moreover, they were restricted to limbic regions, including the medial temporal lobe and amygdala. These cases comprised high amounts of pathological Aβ (plaques) and τ NFT pathology. On the other end of the spectrum, the TDP-43 proteinopathy in FTLD-TDP cases consist of a large abundance of DNs and NCIs, that were detected not only with pTDP-43 antibodies, but also C- and N-t-TDP-43 antibodies (C-t-TDP-43^+^ and N-t-TDP-43^+^, respectively). Moreover, these lesions were widespread in the brain. In addition, in these cases TDP-43 was phosphorylated in S403/404 residues, in addition to S409/S410. Similar to AD^TDP + CTF^ cases, AD^TDP + FL^ cases had high amounts of Aβ and p-τ. However, they comprised an FTLD-TDP-like molecular pattern, which mostly consisted of NCIs, DNs and NFT-like lesions that comprise the full-length protein with phosphorylation of S403/404 in addition to S409/410, and that are widespread in the brain. AD^TDP-^ cases were clinically AD. AD^TDP + CTF^ cases mostly presented a clinical AD phenotype, with some cases presenting svPPA or additional behavioral deficits. Most AD^TDP + FL^ cases presented symptomatic AD, with some cases presenting additional behavioral or language problems and other cases presenting bvFTD. FTLD-TDP cases presented a clinical picture of FTD

We identified distinct molecular patterns of TDP-43 species in neuropathologically-confirmed AD cases. One pattern exhibiting pTDP-43^409/410^, pTDP-43^409^, pTDP-43^403/404^, and non-phosphorylated N- and C-terminal epitopes of TDP-43 indicated the presence of full-length TDP-43 aggregates with a complex phosphorylation pattern including multiple phosphorylation sites. AD cases showing this pattern were referred to as AD^TDP + FL^. This TDP-43 epitope exhibition pattern was also found in FTLD-TDP cases. The other pattern - seen in most p-preAD cases and in AD^TDP + CTF^ cases was restricted to material stained with the anti-pTDP-43^409^ and pTDP-43^409/410^ antibodies, lacking pTDP-43^403/404^ and rarely positive for non-phosphorylated epitopes of TDP-43. Additionally, 19,6% of all AD cases lacked pTDP-43 inclusions and were considered as AD^TDP-^ cases. Accordingly, TDP-43 pathology in AD showed a spectrum ranging from the complete absence of TDP-43 lesions in AD^TDP-^ cases to AD^TDP + CTF^, and finally to AD^TDP + FL^ cases with a molecular pattern similar to FTLD-TDP cases (Fig. 8).

Serines 403/404 and 409/410 have been previously described as TDP-43 sites that are abnormally phosphorylated in the brains of ALS and FTLD-TDP and AD patients [2, 18, 30]. However, it is not yet clear whether pathomechanistic variations are responsible for these different phosphorylation patterns among TDP-43 proteinopathies. Our findings support the hypothesis that phosphorylation at distinct sites of TDP-43 has impact on the molecular type of AD-related TDP-43 pathology and its relation to FTD symptoms. Clinically, 80% of the AD^TDP + CTF^ cases were characterized by typical AD symptoms with leading amnestic and cognitive decline and only 20% of the cases by language problems indicative for PPA. The AD^TDP + FL^ cases presented typical AD symptoms in 63,6% and FTD-like symptoms, such as behavior or language problems in 36,4%.

Morphologically, the majority of TDP-43 lesions in AD^TDP + CTF^ cases consisted of large NCIs, few DNs and NFT-like structures, consistent with previous studies [26, 39]. On the other hand, AD^TDP + FL^ cases displayed abundant DNs and NCIs, as well as NFT-like structures in some cases, which were particularly predominant in temporal and frontal cortices. Of note, we observed no strict association to a specific FTLD-TDP subtype (A-D) as defined by Mackenzie et al [39], considering that there was morphological variability in the AD^TDP + FL^ cases as in FTLD-TDP. Moreover, 4 out of the 11 AD^TDP + FL^ cases did not fit into any FTLD-TDP subtype. These cases displayed large, TDP43-positive NCIs and few DNs scattered among all layers of the temporal cortex and were clinically AD cases. Nevertheless, all AD^TDP + FL^ cases presented a common molecular pattern, which was the focus of our study. Importantly, this pattern was also observed in the typical FTLD-TDP cases. Moreover, Josephs et al. [26] recently studied the morphology of TDP-43 lesions in non-FTLD cases. They identified two morphological signatures for their cases: one related to NCIs and DNs distributed widespread over the brain including frontal cortex (type α), and another related to NFT-associated TDP-43 inclusions, restricted to the medial temporal lobe (type β). Our results corroborate these data in the sense that all AD^TDP + CTF^ and 45,4% of AD^TDP + FL^ cases display type β, whereas the remaining 54,5% of the AD^TDP + FL^ cases present features of both types, α and β, considering that they exhibited not only dystrophic neurites and NCIs in a widespread distribution, but also NFT-associated neuronal inclusions. Thus, we extend this knowledge by distinguishing distinct molecular patterns of TDP-43 species, with the ‘TDP + CTF’ pattern being significantly associated to type β whereas type α features were restricted to a subset of AD^TDP + FL^ cases. Interestingly, these AD^TDP + FL^ cases with a type α TDP-43 pattern were those to show FTD symptoms in 57% of the cases whereas none of our AD^TDP + FL^ cases without type α features, i.e. without frontal or temporal TDP-43 pathology, exhibited signs of FTD. Thus, we can conclude that TDP-43 pathology in AD can cause FTD symptoms when TDP-43 lesions extent into the cortex. This argues strongly in favor of similar underlying processes in FTLD-TDP and AD^TDP + FL^ with cortical TDP-43 pathology, probably to coexisting AD and FTLD-TDP in AD^TDP + FL^ cases. Interestingly, 4 AD^TDP + FL^ cases that were considered as Josephs’ type β were symptomatically AD. In turn, Josephs’ type α was statistically associated with FTD symptoms, which makes it tempting to speculate that type α may play a role in the FTD symptomatology, however more investigation regarding the association of types α and β with clinical symptoms needs to be done.

A few non-AD controls, as well as cognitively normal p-preAD cases also presented TDP-43 proteinopathy, consistent with other studies that observed TDP-43 pathology in cognitively normal individuals [3, 40, 45, 67], which was associated with PART [28, 68]. The TDP-43 molecular pattern in these cases was similar to that seen in AD^TDP + CTF^ cases, as well as the morphology of these lesions, which consisted of NFT-associated material, reflecting Josephs’ type β TDP-43 pathology.

Overall, these results point to a potential difference in the mechanism of TDP-43 proteinopathy between AD^TDP + CTF^ and AD^TDP + FL^, with the latter similar to that of FTLD-TDP. Our results also indicate that C-terminal fragments (CTFs) of TDP-43 are enriched in AD^TDP + CTF^, consistent with other studies [22]. Such mechanisms could be related to different kinases phosphorylating serines 403/404 and 409/410, considering that the pattern of phosphorylation at these sites distinguishes these groups of cases. Alternatively, genetic differences could also explain the distinct neuropathological TDP-43 patterns [26, 47]. Interestingly, one case in the AD^TDP + FL^ group had a mutation in an FTLD-TDP causing gene, i.e. in the *C9ORF72* gene [13, 55]. This supports our interpretation of the ‘TDP + FL’ pattern as possibly biologically linked to FTLD-TDP at least in some of these cases probably exhibiting co-existing AD and FTLD-TDP. On the other hand, the *C9ORF72* mutation has been previously found in a very low amount of AD cases [17].

A third explanation for the different patterns of TDP-43 pathology in AD cases could be that TDP-43 plays different roles in these patients. In AD, the accumulation of presumably N-terminal truncated pTDP-43^409/410^ may represent a secondary event, maybe co-seeded by τ or Aβ, as hypothesized by others [12, 20, 36]. An argument supporting this hypothesis is that TDP-43 pathology in our control cases occurred in the same anatomical regions, in which PART-lesions (NFTs and neuropil threads) were co-existing. The morphological appearance of the TDP-43 lesions in AD^TDP + CTF^ cases as NFTs may also argue for a secondary phenomenon induced by the underlying τ pathology [1, 60]. Non-specific detection of NFTs by anti-TDP-43^409/410^ antibodies has also been discussed [38]. However, in our study three different antibodies against pTDP-43^409/410^, including a monoclonal antibody, labelled NFTs, arguing against non-specific staining. Furthermore, all of our AD^TDP + CTF^ cases had high amounts of τ protein pathology in the frontal cortex, but no anti-pTDP-43^409/410^ or anti-pTDP-43^409^ positive material, which also argues against non-specific labelling of anti-pTDP-43 antibodies in the hippocampus or the amygdala. This is strengthened by the reports of other authors that NFT-like material can be detected with non-phosphorylated anti-TDP-43 antibodies [1, 26], which suggests a strong association between τ and TDP-43. On the other hand, one may speculate that TDP-43 acts as the primary pathology in AD^TDP + FL^ cases, similarly to FTLD-TDP. In light of these arguments, it is tempting to speculate that both secondary accumulation of TDP-43 and primary TDP-43 pathology may occur in AD cases: Secondary accumulation of pTDP43^409/410^ and pTDP-43^409^-positive material in AD^TDP + CTF^ cases and primary development of TDP-43 aggregates in AD^TDP + FL^ cases.

There has been growing evidence regarding the existence of concomitant neuropathologies, in which a neurodegenerative disease might have additional aggregated proteins besides the primary pathology, accumulating as co-pathologies [4, 15, 32, 57, 63]. AD^TDP + FL^ cases described in this study appear to be an example of this, as they present a molecular pattern of TDP-43 pathology similar to that seen in FTLD-TDP cases, as well as histological full-blown AD pathology (as observed with antibodies against Aβ and τ). Furthermore, 7 of these 11 cases presented a morphological and topographic distribution of TDP-43 compatible with one of the FTLD-TDP subtypes (A-C). This stresses the importance of considering multiple pathologies contributing to the development of dementia, as seen in previous studies [52]. The recent consensus work regarding LATE might also be important in this context [47]. LATE-NC was described as the presence of TDP-43 pathology in the limbic areas of elderly, with or without co-existing AD pathology. As seen here, a few non-AD controls, the majority of p-preAD and all AD^TDP + CTF^, as well as AD^TDP + FL^ cases fit into this new classification (Table 2, Additional file 1-Table A1). The controls, p-preAD, and AD^TDP + CTF^ cases that exhibited LATE-NC, mostly presented LATE-NC stage 2, which means the TDP-43 pathology is predominantly present in amygdala and hippocampus. On the other hand, 54,5% of AD^TDP + FL^ cases had additional TDP-43 lesions in the frontal cortex, indicative for LATE-NC stage 3. Of note, 19,6% of all neuropathologically-confirmed AD cases did not present TDP-43 pathology (i.e LATE-NC) at all, consistent with other studies [44, 47]. Interestingly, the p-preAD cases in our cohort showed a high prevalence of TDP-43 pathology, reflected in the 68,7% of cases with LATE-NC stage 1 or stage 2. Given the hierarchical detectability of first pTDP-43^409^ and pTDP-43^409/410^ in cases with LATE-NC stages 1–2 and second other TDP-43 epitopes in AD^TDP + FL^ cases with LATE-NC stages 2 and 3, one could hypothesize that TDP-43 aggregates in AD undergo maturation changes similar to Aβ aggregates [56]. An argument against this hypothesis is that both AD-related τ and Aβ pathology showed late stages of AD pathology in AD^TDP-^, AD^TDP + CTF^ and AD^TDP + FL^ cases, and that there was no significant difference in age among these groups. Secondly, the identification of one AD^TDP + FL^ case with a *C9ORF72* gene mutation strongly suggests a specific FTLD-like influence of TDP-43 pathology in at least a subset of these cases and, thereby, also argues against a simple maturation process of TDP-43 aggregates that distinguishes AD^TDP + CTF^ and AD^TDP + FL^ cases. In our opinion, it is therefore likely that the different molecular patterns of TDP-43 pathology among AD cases may constitute subtypes of LATE-NC or alternatively argue in favor of spectrum of AD ranging from AD^TDP-^ without TDP-43 pathology to AD^TDP + FL^ with probably coexisting FTLD pathology. However, the LATE concept is controversial and further investigation addressing the morphology of these lesions and its relationship to the molecular and clinical patterns is needed. Nevertheless, if these patterns constitute subtypes of LATE-NC, the umbrella term ‘LATE-NC’ could include different molecular types of TDP-43 lesions that give rise to the hypothesis that LATE-NC covers biologically different lesions/diseases: 1. TDP-43 pathology with predominance of phosphorylated C-terminal fragments of TDP-43 as in AD^TDP + CTF^, 2. full-length TDP-43 pathology as in AD^TDP + FL^ similar as in FTLD-TDP, 3. hippocampal sclerosis, and maybe others that we are not yet aware of. Whether the full-length TDP-43 predominant pattern in AD^TDP + FL^ cases distinguishes co-existing FTLD-TDP pathology from the C-terminal fragment-predominant lesions, needs to be clarified in the future.

Importantly, the ‘TDP + CTF’ molecular pattern (observed in some non-AD, most p-preAD and AD^TDP + CTF^ cases) was significantly associated with typical AD symptoms, whereas the ‘TDP + FL’ molecular pattern (observed in AD^TDP + FL^ and FTLD-TDP cases) was associated with symptoms of the FTD spectrum, especially in those AD^TDP + FL^ cases that exhibit TDP-43 lesions in the frontal and/or temporal neocortex, i.e. representing the type α TDP-43 distribution pattern (Fig. 8). This suggests that the molecular profile of TDP-43 species in these cases together with the cortical distribution of the lesions influences the clinical presentation of the disease to a certain degree. However, typical AD symptoms were seen in more than 50% of the cases in both AD subgroups. This indicates that the ‘TDP + FL’ molecular pattern had impact on less than 50% of the cases with various phenotypes typical of the FTD spectrum (36,4%). Interestingly, in the AD^TDP + CTF^ group, less than 20% of the cases showed other symptoms besides cognitive deficits and executive dysfunction. These were related to language alterations in 16,6% of the cases, with only 1 case showing additional behavioral changes.

AD and LATE-NC can occur in the same individuals and both are associated with cognitive decline and amnestic symptoms, whereas FTLD-TDP is characterized by alterations in behavior and/or language [47]. It has been shown that the co-existence of LATE-NC and AD pathology is associated with clinically more severe symptoms than pure AD (in the absence of TDP-43 pathology, i.e. LATE-NC) [37]. Indeed, TDP-43 has been demonstrated to worsen cognition in aged individuals [29, 31, 46, 53], probably due to a synergistic effect with τ protein [37, 60]. The AD^TDP + FL^ cases in our cohort presented a broad range of clinical phenotypes, from a primary amnestic deficit (typical for AD) to personality and language changes (typical for FTD). This has impact on the clinical differential diagnosis of degenerative dementing disorders. On the one hand, cases presenting a clinical phenotype of the FTLD spectrum may display significant levels of AD pathology, as seen in our results, which might be associated with positive AD biomarkers. This is also valid for those AD^TDP + CTF^ cases that presented a svPPA phenotype. On the other hand, cases exhibiting a typical AD clinical phenotype and biomarker profile might present a widespread FTLD-TDP-like TDP-43 pathology and molecular pattern as well. Thus, AD-related treatments might possibly be less effective than expected in these cases. This may also have implications for clinical praxis, in the sense that the screening for AD biomarkers in cases with a clinical picture of FTD might be relevant. This would avoid missing AD lesions in AD^TDP + FL^ cases. Overall, our data highlight the relevance of underlying pathologies for the diagnosis and treatment of patients.

One limitation of this study is that the post-mortem intervals of the cases in our cohort were variable (24 to 120 h). Furthermore, the fixation time was also variable (2–4 weeks) due to the fact that the sample consists of several hospital-based cohorts, in which the tissue was acquired at different time-points. However, we did not observe obvious differences in the TDP-43 staining quality among our cases, indicating that these limitations had no significant impact on our results. Another limitation is that there is not a standard TDP-43 antibody recommended to characterize TDP-43 proteinopathies. To overcome this problem, we chose several commercially available, phosphorylation dependent and independent antibodies to provide reliable results. In addition, the number of AD^TDP + FL^ and FTLD-TDP cases was limited (eleven and ten, respectively). The difference in age distribution among the groups and the use of hospital-based cohorts may be considered as another limitation of this study. Logistic regressions controlling for age at death showed that especially controls and FTLD-TDP cases were younger compared to the AD group. This could be due to the low number of cases. However, the younger age of death of control cases free of any AD pathology can be expected, as in older individuals we often observe AD-related pathology. Therefore, only few cases at higher ages may serve as non-AD controls [8, 62], leading to the selection of younger cases as controls compared to older ones with full-blown AD. To take this age difference into account in our analyses, we included age and sex in logistic regression models as additional independent variables. Finally, the low number of cases with distinct clinical phenotypes in the AD^TDP + FL^ group is another limitation. To determine the impact of this limitation, we performed a post-hoc power analysis for our data and obtained a statistical power of ≥80% when analyzing the different TDP-43 antibody patterns among the disease groups, as well as other neuropathological parameters (i.e.: Braak NFT-stages, AβMTL phases, CERAD score, etc). For the clinical parameters, we had a statistical power of 48–87% indicating that the interpretation of our results is solid for the neuropathological groups and parameters. Conclusions about clinical phenotypes/parameters, on the other hand, must be considered with caution. Since we did not have a community-based cohort of cases, we cannot exclude influence of the hospital-based sampling on our results. However, the prevalence of TDP-43 pathology in elderly individuals of community-based samples in the literature showed a similarly high prevalence of TDP pathology in the brain [24, 40].

In conclusion, we were able to identify molecular differences in TDP-43 pathological lesions, distinguishing distinct patterns of TDP-43 pathology in neuropathologically-confirmed AD cases, one of which being similar to the pattern observed in FTLD-TDP. These patterns differed in the prevalence of truncated and non-truncated TDP-43 species and of distinct phosphorylation epitopes. Furthermore, we showed that these patterns were associated with the frequency of FTD symptoms. These differences may have an impact for future diagnostic algorithms and treatment of patients with clinical signs of dementia. Therefore, underlying pathologies need to be considered when diagnosing and consequently treating demented patients. Whether these molecular patterns of TDP-43 pathology represent types of LATE-NC and, thereby, are features of one disease entity (LATE) or whether they represent a spectrum of late-life neuropathologies in AD ranging from AD^TDP-^ to AD^TDP + CTF^ and finally AD^TDP + FL^ with molecular similarities to FTLD-TDP needs to be explored in the future.

## Supplementary information

**Additional file 1: Figure A1.** - pTDP-43^409/410^ species are predominant in p-preAD and AD^TDP + CTF^ whereas AD^TDP + FL^ and FTLD-TDP display positivity for all pTDP-43 epitopes in several regions. Immunohistochemistry of a non-AD, p-preAD, AD^TDP + CTF^, AD^TDP + FL^ and FTLD-TDP case in dentate gyrus (a,d,h), temporal (b,e,i) and frontal (c,f,j) cortices with (a1-c5) pTDP-43^409/410^ (clone 1D3), (d1-f5) pTDP-43^409^ and (h1-j5) pTDP-43^403/404^, displaying lesions (arrowheads). P-preAD and AD^TDP + CTF^ cases displayed mostly NFT-like inclusions with pTDP-403^409^ in temporal cortex (e2-e3). AD^TDP + FL^ and FTLD-TDP cases showed NCIs in the DG (a4-a5 respectively, arrowheads), NCIs and DNs in temporal cortex (b4-b5 respectively, arrowheads) and DNs in frontal cortex (c4-c5 respectively, arrowheads) when stained with all pTDP-43 antibodies. AD^TDP-^ cases were not included in this figure because no TDP-43 inclusions were observed. Scale bar = 50 μm. **Figure A2.** – AD^TDP + FL^ and FTLD-TDP display inclusions positive for non-phosphorylated TDP-43, but not p-preAD or AD^TDP + CTF^ cases. Immunohistochemistry of a non-AD, p-preAD, AD^TDP + CTF^ AD^TDP + FL^ and FTLD-TDP case in dentate gyrus (a, d), temporal (b, e) and frontal (c, f) cortices with C- and N-t TDP-43, displaying cytoplasmic lesions (arrowheads) and nuclear clearance (arrows). AD^TDP + FL^ and FTLD-TDP cases also displayed NCIs in the DG (d4-d5 respectively, arrowheads) with clearance of normal C-t-TDP-43 from the nucleus (a4-a5, arrows), DNs in the temporal cortex (b4-b5 respectively, arrowheads) and DNs in frontal cortex (c4-c5 respectively, arrowheads) when stained with C-t-TDP-43. Finally, AD^TDP + FL^ and FTLD-TDP cases showed NCIs in the DG (d4-d5 respectively, arrowheads) with clearance of normal N-t-TDP-43 from the nucleus (arrows), DNs in the temporal cortex (e4-e5 respectively, arrowheads) and DNs in the frontal cortex (f4-f5 respectively, arrowheads) when stained with N-t-TDP-43. AD^TDP-^ cases were not included in this figure because no TDP-43 inclusions were observed. Scale bar = 50 μm. **Figure A3.** - Percentage of positive cases for DNs, NCIs, NIIs or NFT-like lesions detected with pTDP-43^409/410^, pTDP-43^409^, pTDP-43^403/404^, C- and N-t-TDP-43 in (a) amygdala, (b) NBM, (c) CA4, (d) CA3/2, (e) subiculum, (f) entorhinal cortex, (g) frontal cortex and (h) occipital cortex. Grouping of cases was done according to the neuropathological criteria for non-AD (*n* = 20), p-preAD (*n* = 16), AD^TDP-^ (*n* = 10), AD^TDP + CTF^ (*n* = 30) and FTLD-TDP cases (n = 10). AD^TDP + FL^ cases (*n* = 11) were considered as neuropathologically-confirmed AD cases with a molecular TDP-43 pattern similar to that of FTLD-TDP. **Figure A4.** – Clinical grouping of our cohort shows significant differences within the different antibodies in the symptomatic AD group. Quantifications of positive cases for DNs, NCIs, NIIs or NFT-like lesions detected with pTDP-43^409/410^, pTDP-43^409^, pTDP-43^403/404^, C- and N-t-TDP-43 in (a) amygdala, (b) NBM, (c) CA4, (d) CA3/2, (e) subiculum, (f) entorhinal cortex, (g) frontal cortex and (h) occipital cortex. Non-parametric, Friedman test for related samples with Bonferroni correction for multiple testing was used to compare the amount of positive cases of each TDP-43 antibody. Each group was analyzed separately, *p < 0.05, **p < 0.01. Non-demented cases included the cases from the non-AD and p-preAD neuropathological groups (*n* = 36). SympAD cases referred to cases with exclusive AD symptoms (including AD^TDP + CTF^, AD^TDP-^ and some AD^TDP + FL^ cases, *n* = 40), AD/FTD refer to cases with both AD and FTD signs (*n* = 5) and FTD group represents cases with an exclusive FTD presentation (*n* = 15). **Table A1 -** Human cases used in this study. Abbreviations; NA: not applicable; na: not assessed; m: male; f: female; PART: primary age-related tauopathy; CAA: cerebral amyloid angiopathy; AGD: argyrophilic grain disease; LBD: Lewy body disease. **Table A2**- List of antibodies used in this study. Abbreviations; IHC: immunohistochemistry, IF: immunofluorescence; Rb: rabbit, Ms.: mouse; ND: non-determined, N-t: N-terminal, C-t: C-terminal. **Table A3**- percentage of positive cases for pTDP-43^409/410^. **Table A4**- percentage of positive cases for pTDP-43^409^. **Table A5**- percentage of positive cases for pTDP-43^403/404^. **Table A6**- percentage of positive cases for C-t-TDP-43. **Table A7**- percentage of positive cases for N-t-TDP-43. **Table A8 a –** Binary logistic regression addressing the differences between AD^TDP + CTF^ vs. AD^TDP + FL^ regarding Braak NFT-staging, when controlled for age and sex. **b –** Binary logistic regression addressing the differences between AD^TDP + CTF^ vs. AD^TDP-^regarding Braak NFT-staging, when controlled for age and sex. **c –** Binary logistic regression addressing the differences between AD^TDP + FL^ vs. AD^TDP-^regarding Braak NFT-staging, when controlled for age and sex. * after Bonferroni correction for multiple testing. **Table A9 a –** Binary logistic regression addressing the differences between AD^TDP + CTF^ vs. AD^TDP + FL^ regarding AβMTL phase, when controlled for age and sex. **b –** Binary logistic regression addressing the differences between AD^TDP + CTF^ vs. AD^TDP-^ regarding AβMTL phase, when controlled for age and sex. **c –** Binary logistic regression addressing the differences between AD^TDP + FL^ vs. AD^TDP-^ regarding AβMTL phase, when controlled for age and sex. * after Bonferroni correction for multiple testing. **Table A10 a –** Binary logistic regression addressing the differences between AD^TDP + CTF^ vs. AD^TDP + FL^ regarding CERAD score, when controlled for age and sex. **b –** Binary logistic regression addressing the differences between AD^TDP + CTF^ vs. AD^TDP-^ regarding CERAD score, when controlled for age and sex. **c –** Binary logistic regression addressing the differences between AD^TDP + FL^ vs. AD^TDP-^ regarding CERAD score, when controlled for age and sex. * after Bonferroni correction for multiple testing. **Table A11 a –** Binary logistic regression addressing the differences between AD^TDP + CTF^ vs. AD^TDP + FL^ regarding NIA-AA degree of AD pathology, when controlled for age and sex. **b –** Binary logistic regression addressing the differences between AD^TDP + CTF^ vs. AD^TDP-^ regarding NIA-AA degree of AD pathology, when controlled for age and sex. **c –** Binary logistic regression addressing the differences between AD^TDP + FL^ vs. AD^TDP-^ regarding NIA-AA degree of AD pathology, when controlled for age and sex. * after Bonferroni correction for multiple testing. **Table A12** – Binary logistic regression addressing the association of the TDP + CTF pattern with types α and β, controlled for age at death and sex. Cases with type α + β were considered as positive for type α as well as for type β, respectively. **Table A13** – Binary logistic regression addressing the association of the TDP + FL pattern with types α and β, controlled for age at death and sex. Cases with type α + β were considered as positive for type α as well as for type β, respectively. **Table A14 –***P* values of comparisons between TDP-43 antibodies for non-demented cases, *n* = 36. Friedman test with Bonferroni correction for multiple testing. Abbreviations; NBM: basal nucleus of Meynert, DG: dentate gyrus. **Table A15 –** P values of comparisons between TDP-43 antibodies groups for symptomatic AD cases, *n* = 33. Friedman test with Bonferroni correction for multiple testing. Abbreviations; NBM: basal nucleus of Meynert, DG: dentate gyrus. **Table A16 –** P values of comparisons between TDP-43 antibodies groups for AD/FTD cases, *n* = 5. Friedman test with Bonferroni correction for multiple testing. Abbreviations; NBM: basal nucleus of Meynert, DG: dentate gyrus. **Table A17 –** P values of comparisons between TDP-43 antibodies groups for FTD cases, *n* = 15. Friedman test with Bonferroni correction for multiple testing. Abbreviations; NBM: basal nucleus of Meynert, DG: dentate gyrus. **Table A18 –** Multinomial logistic regressions addressing the differences between disease groups, for pTDP^409/410^, in CA1 region. Symptomatic AD cases were considered as the reference category for comparison. Fields were marked with “-” when the software could not assess the statistical value due to overflow. **Table A19 –** Multinomial logistic regressions addressing the differences between disease groups, for pTDP^409^, in CA1 region. Symptomatic AD cases were considered as the reference category for comparison. Fields were marked with “-” when the software could not assess the statistical value due to overflow. **Table A20 –** Multinomial logistic regressions addressing the differences between disease groups, for pTDP^403/404^, in CA1 region. Symptomatic AD cases were considered as the reference category for comparison. Fields were marked with “-” when the software could not assess the statistical value due to overflow. **Table A21 –** Multinomial logistic regressions addressing the differences between disease groups, for C-t TDP-43, in CA1 region. Symptomatic AD cases were considered as the reference category for comparison. Fields were marked with “-” when the software could not assess the statistical value due to overflow. **Table A22 –** Multinomial logistic regressions addressing the differences between disease groups, for N-t TDP-43, in CA1 region. Symptomatic AD cases were considered as the reference category for comparison. Fields were marked with “-” when the software could not assess the statistical value due to overflow. **Table A23** Binary logistic regression addressing the association of Josephs’ TDP-43 pathology type α with FTD symptoms, controlled for age at death and sex. Cases with type α + β were considered as positive for type α as well as for type β, respectively. **Table A24** – Binary logistic regression addressing the association of Josephs’ TDP-43 pathology type β with FTD symptoms, controlled for age at death and sex. Fields were marked with “-” when the software could not assess the statistical value due to overflow. Cases with type α + β were considered as positive for type α as well as for type β, respectively. **Table A25** Binary logistic regression addressing the association of FTD symptoms with TDP + CTF pattern, controlled for age at death and sex. Only demented cases were considered. **Table A26** – Binary logistic regression addressing the association of typical AD symptoms with TDP + FL pattern, controlled for age at death and sex. Only demented cases were considered.

## Data Availability

The anonymized datasets used and/or analyzed during the current study are stored in UZ/KU-Leuven network drives and available from the corresponding author on reasonable request.

## Abbreviations

TDP-43: Transactive Response DNA-Binding Protein
pTDP-43: phosphorylated TDP-43
Aβ: Amyloid-beta protein
p-τ: phosphorylated tau protein
AD: Alzheimer’s Disease
p-preAD: pathologically-defined preclinical AD
FTLD-TDP: Frontotemporal lobar degeneration with TDP-43 inclusions
NCIs: Neuronal cytoplasmic inclusions
NDNs: Dystrophic neurites
NFTs: Neurofibrillary tangles
AD^TDP-^: Neuropathologically-confirmed AD cases without TDP-43 pathology
AD^TDP + CTF^: Neuropathologically-confirmed AD cases with pTDP-43 pathology but no non-phosphorylated or pTDP-403^403/404^ pathology
AD^TDP + FL^: Neuropathologically-confirmed AD cases with TDP-43 pathology positive for all markers in this study
LATE: Limbic-predominant age-related TDP-43 encephalopathy
LATE-NC: Limbic-predominant age-related TDP-43 encephalopathy related neuropathological changes
FTD: Frontotemporal dementia
svPPA: semantic variant of primary progressive aphasia
PSP: Progressive supranuclear palsy
