# Supplementary material for: Distinct molecular patterns of TDP-43 pathology in Alzheimer’s disease: relationship with clinical phenotypes
Source: Acta Neuropathol Commun. 2020 Apr 29;8:61. doi: 10.1186/s40478-020-00934-5 (PMC7189555; doi:10.1186/s40478-020-00934-5)
Supplement: Supplementary file 1 — Additional file 1: Figure A1. - pTDP-43409/410 species are predominant in p-preAD and ADTDP + CTF whereas ADTDP + FL and FTLD-TDP display positivity for all pTDP-43 epitopes in several regions. Immunohistochemistry of a non-AD, p-preAD, ADTDP + CTF, ADTDP + FL and FTLD-TDP case in dentate gyrus (a,d,h), temporal (b,e,i) and frontal (c,f,j) cortices with (a1-c5) pTDP-43409/410 (clone 1D3), (d1-f5) pTDP-43409 and (h1-j5) pTDP-43403/404, displaying lesions (arrowheads). P-preAD and ADTDP + CTF cases displayed mostly NFT-like inclusions with pTDP-403409 in temporal cortex (e2-e3). ADTDP + FL and FTLD-TDP cases showed NCIs in the DG (a4-a5 respectively, arrowheads), NCIs and DNs in temporal cortex (b4-b5 respectively, arrowheads) and DNs in frontal cortex (c4-c5 respectively, arrowheads) when stained with all pTDP-43 antibodies. ADTDP- cases were not included in this figure because no TDP-43 inclusions were observed. Scale bar = 50 μm. Figure A2. – ADTDP + FL and FTLD-TDP display inclusions positive for non-phosphorylated TDP-43, but not p-preAD or ADTDP + CTF cases. Immunohistochemistry of a non-AD, p-preAD, ADTDP + CTF ADTDP + FL and FTLD-TDP case in dentate gyrus (a, d), temporal (b, e) and frontal (c, f) cortices with C- and N-t TDP-43, displaying cytoplasmic lesions (arrowheads) and nuclear clearance (arrows). ADTDP + FL and FTLD-TDP cases also displayed NCIs in the DG (d4-d5 respectively, arrowheads) with clearance of normal C-t-TDP-43 from the nucleus (a4-a5, arrows), DNs in the temporal cortex (b4-b5 respectively, arrowheads) and DNs in frontal cortex (c4-c5 respectively, arrowheads) when stained with C-t-TDP-43. Finally, ADTDP + FL and FTLD-TDP cases showed NCIs in the DG (d4-d5 respectively, arrowheads) with clearance of normal N-t-TDP-43 from the nucleus (arrows), DNs in the temporal cortex (e4-e5 respectively, arrowheads) and DNs in the frontal cortex (f4-f5 respectively, arrowheads) when stained with N-t-TDP-43. ADTDP- cases were not included in this figure [file 40478_2020_934_MOESM1_ESM.docx]

Additional File 1 to:

**Distinct molecular patterns of TDP-43 pathology in Alzheimer’s Disease: relationship with clinical phenotypes**

Sandra Tomé^1^, Rik Vandenberghe^2,3^, Simona Ospitalieri^1^, Evelien Van Schoor^1,4^, Thomas Tousseyn^5,6^, Markus Otto^7^, Christine A.F. von Arnim^7,8^, Dietmar Rudolf Thal^1,6^.

^1^Department of Imaging and Pathology - Laboratory of Neuropathology, and Leuven Brain Institute, KU-Leuven, Leuven, Belgium

^2^Department of Neurosciences – Laboratory of Cognitive Neurology, KU- Leuven, Leuven, Belgium

^3^Department of Neurology, UZ Leuven, Leuven, Belgium

^4^Department of Neurosciences – Laboratory for Neurobiology, KU-Leuven and Center for Brain & Disease Research, VIB, Leuven, Belgium

^5^Department of Imaging and Pathology - Translational Cell and Tissue Research Unit, KU-Leuven, Belgium

^6^Department of Pathology, UZ Leuven, Leuven, Belgium

^7^Department of Neurology, Ulm University, Ulm, Germany

^8^Department of Geriatrics, Göttingen University, Germany

**
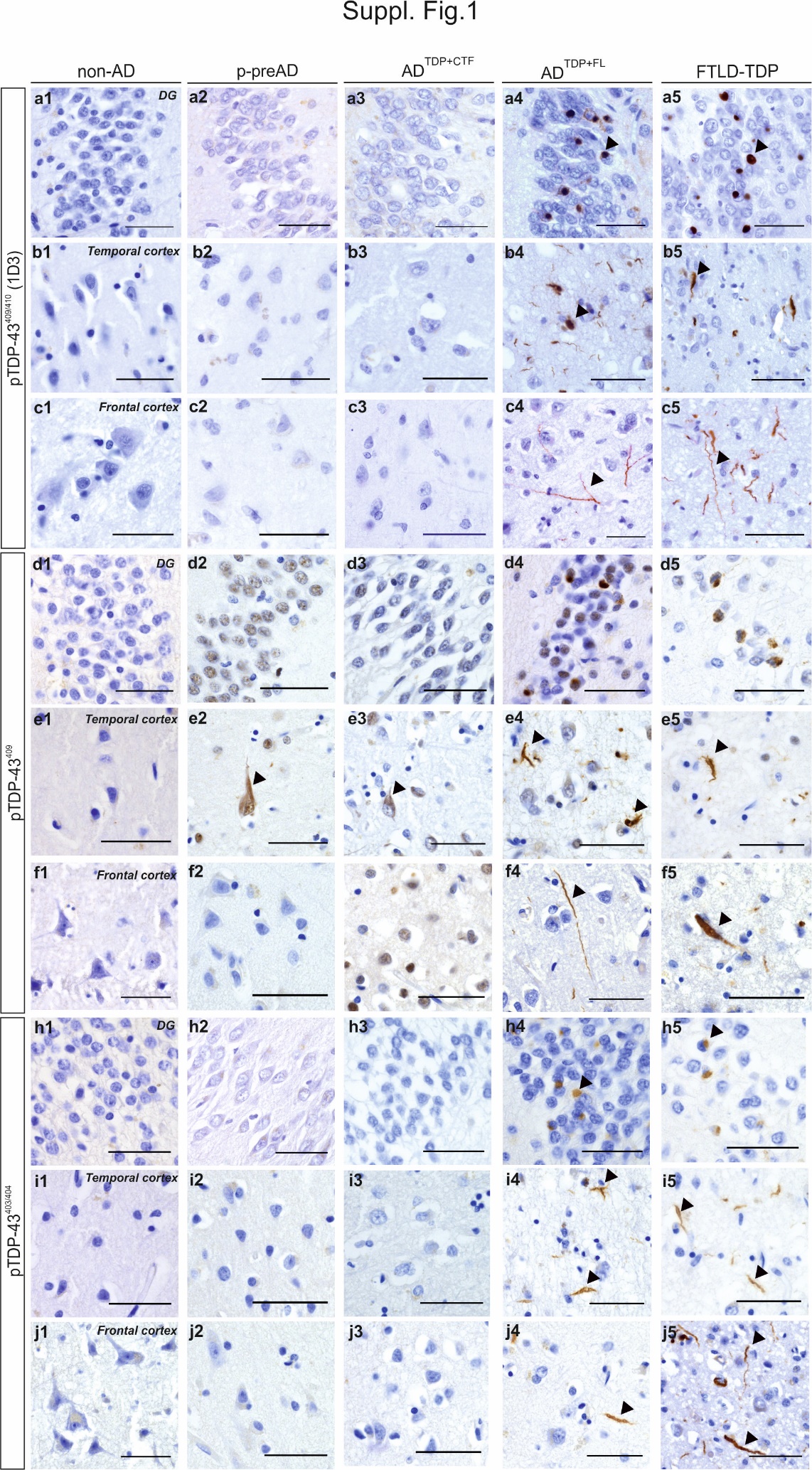
**

**Figure A1** - pTDP-43^409/410^ species are predominant in p-preAD and AD^TDP+CTF^ whereas AD^TDP+FL^ and FTLD-TDP display positivity for all pTDP-43 epitopes in several regions. Immunohistochemistry of a non-AD, p-preAD, AD^TDP+CTF^, AD^TDP+FL^ and FTLD-TDP case in dentate gyrus (a,d,h), temporal (b,e,i) and frontal (c,f,j) cortices with (a1-c5) pTDP-43^409/410^ (clone 1D3), (d1-f5) pTDP-43^409^ and (h1-j5) pTDP-43^403/404^, displaying lesions (arrowheads). P-preAD and AD^TDP+CTF^ cases displayed mostly NFT-like inclusions with pTDP-403^409^ in temporal cortex (e2-e3). AD^TDP+FL^ and FTLD-TDP cases showed NCIs in the DG (a4-a5 respectively, arrowheads), NCIs and DNs in temporal cortex (b4-b5 respectively, arrowheads) and DNs in frontal cortex (c4-c5 respectively, arrowheads) when stained with all pTDP-43 antibodies. AD^TDP-^ cases were not included in this figure because no TDP-43 inclusions were observed. Scale bar = 50 µm.

**
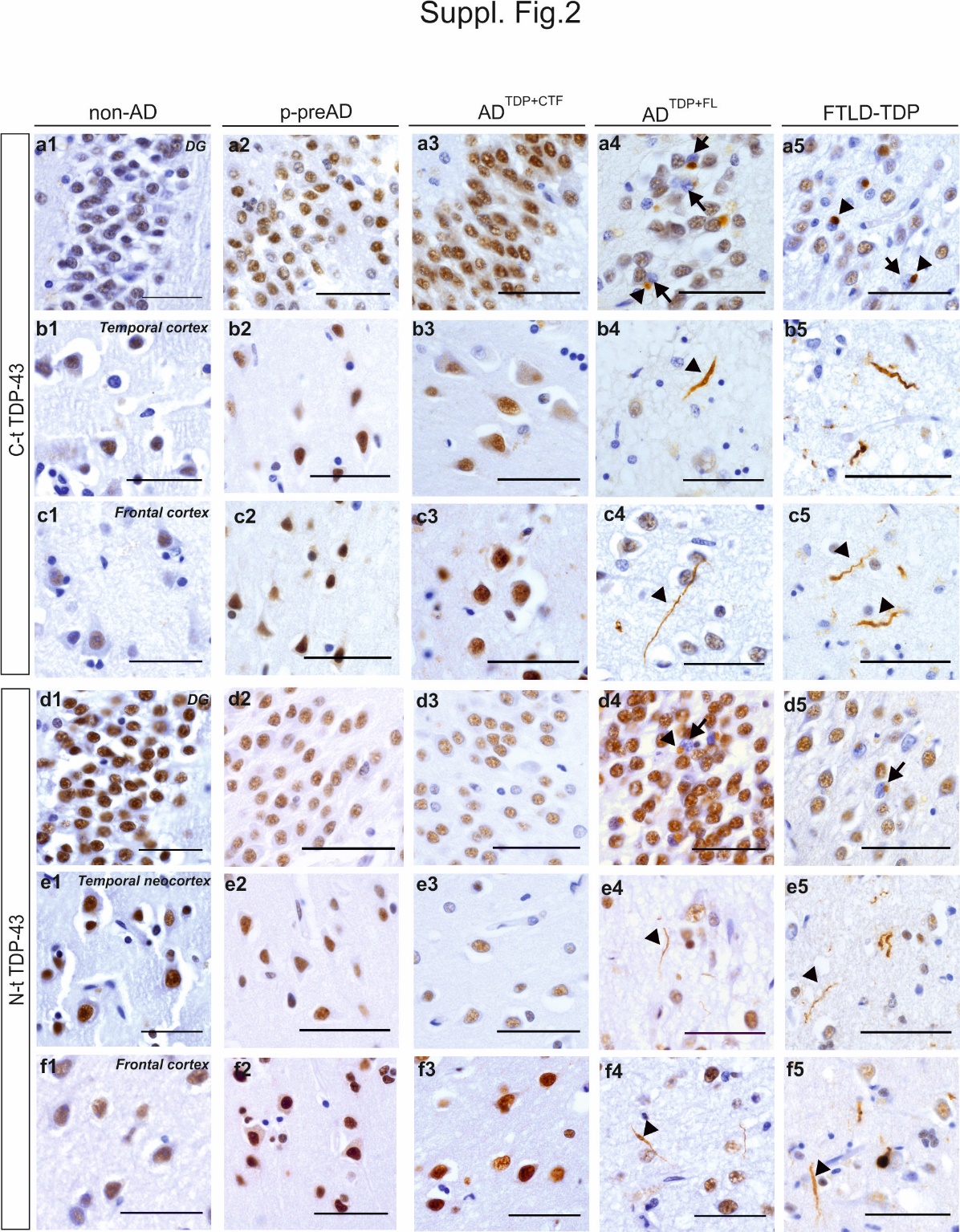
**

**Figure A2** – AD^TDP+FL^ and FTLD-TDP display inclusions positive for non-phosphorylated TDP-43, but not p-preAD or AD^TDP+CTF^ cases. Immunohistochemistry of a non-AD, p-preAD, AD^TDP+CTF^ AD^TDP+FL^ and FTLD-TDP case in dentate gyrus (a, d), temporal (b, e) and frontal (c, f) cortices with C- and N-t TDP-43, displaying cytoplasmic lesions (arrowheads) and nuclear clearance (arrows). AD^TDP+FL^ and FTLD-TDP cases also displayed NCIs in the DG (d4-d5 respectively, arrowheads) with clearance of normal C-t-TDP-43 from the nucleus (a4-a5, arrows), DNs in the temporal cortex (b4-b5 respectively, arrowheads) and DNs in frontal cortex (c4-c5 respectively, arrowheads) when stained with C-t-TDP-43. Finally, AD^TDP+FL^ and FTLD-TDP cases showed NCIs in the DG (d4-d5 respectively, arrowheads) with clearance of normal N-t-TDP-43 from the nucleus (arrows), DNs in the temporal cortex (e4-e5 respectively, arrowheads) and DNs in the frontal cortex (f4-f5 respectively, arrowheads) when stained with N-t-TDP-43. AD^TDP-^ cases were not included in this figure because no TDP-43 inclusions were observed. Scale bar = 50 µm.


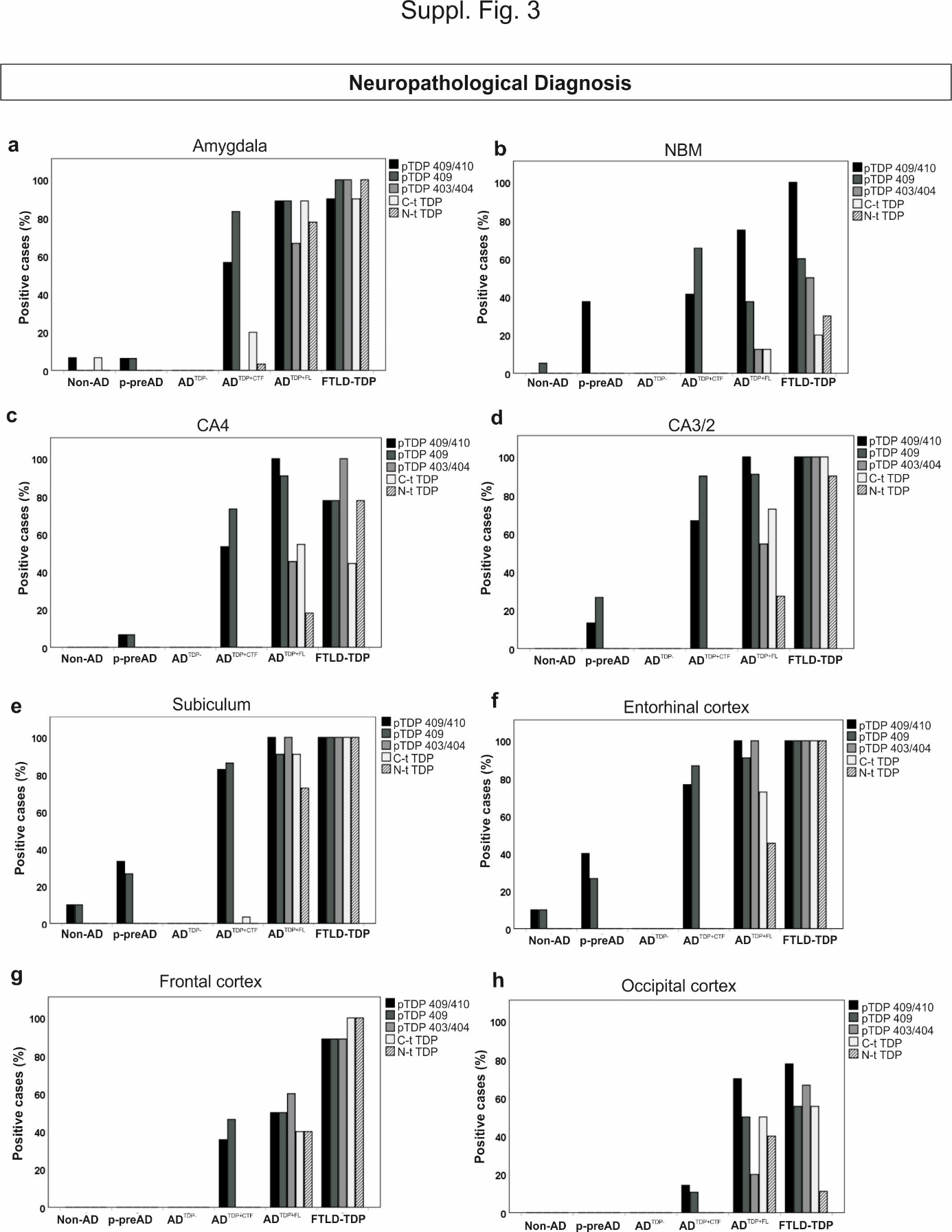


**Figure A3** - Percentage of positive cases for DNs, NCIs, NIIs or NFT-like lesions detected with pTDP-43^409/410^, pTDP-43^409^, pTDP-43^403/404^, C- and N-t-TDP-43 in (a) amygdala, (b) NBM, (c) CA4, (d) CA3/2, (e) subiculum, (f) entorhinal cortex, (g) frontal cortex and (h) occipital cortex. Grouping of cases was done according to the neuropathological criteria for non-AD (n=20), p-preAD (n=16), AD^TDP-^ (n=10), AD^TDP+CTF^ (n=30) and FTLD-TDP cases (n=10). AD^TDP+FL^ cases (n=11) were considered as neuropathologically-confirmed AD cases with a molecular TDP-43 pattern similar to that of FTLD-TDP.

**
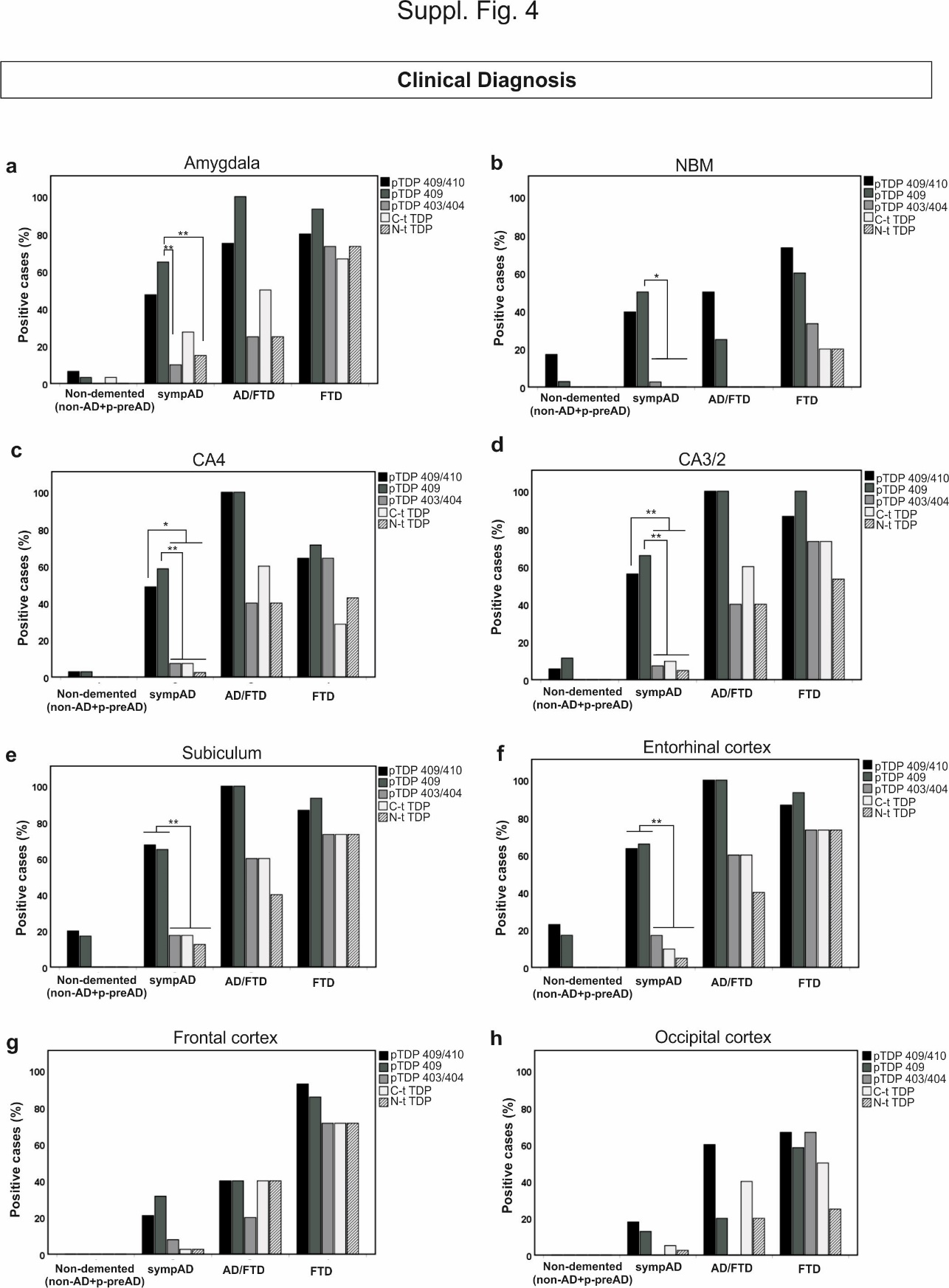
**

**Figure A4** – Clinical grouping of our cohort shows significant differences within the different antibodies in the symptomatic AD group. Quantifications of positive cases for DNs, NCIs, NIIs or NFT-like lesions detected with pTDP-43^409/410^, pTDP-43^409^, pTDP-43^403/404^, C- and N-t-TDP-43 in (a) amygdala, (b) NBM, (c) CA4, (d) CA3/2, (e) subiculum, (f) entorhinal cortex, (g) frontal cortex and (h) occipital cortex. Non-parametric, Friedman test for related samples with Bonferroni correction for multiple testing was used to compare the amount of positive cases of each TDP-43 antibody. Each group was analyzed separately, *p<0.05, **p<0.01. Non-demented cases included the cases from the non-AD and p-preAD neuropathological groups (n=36). SympAD cases referred to cases with exclusive AD symptoms (including AD^TDP+CTF^, AD^TDP-^ and some AD^TDP+FL^ cases, n=40), AD/FTD refer to cases with both AD and FTD signs (n=5) and FTD group represents cases with an exclusive FTD presentation (n=15).

**Table A1 -** Human cases used in this study. Abbreviations; NA: not applicable; na: not assessed; m: male; f: female; PART: primary age-related tauopathy; CAA: cerebral amyloid angiopathy; AGD: argyrophilic grain disease; LBD: Lewy body disease.

| **Case no.** | **Age** | **Sex** | **Neuropathological diagnosis** | **Clinical diagnosis** | **Mutation** | **Braak NFT stage** [1] | **AβMTL phase** [8] | **CERAD score** [5] | **NIA-AA degree** [2] | **LATE-NC stage** [7] | **TDP-43 subtype** [3] | **CDR** [4, 6] |
| --- | --- | --- | --- | --- | --- | --- | --- | --- | --- | --- | --- | --- |
| 1 | 61 | m | Non-AD | Non-demented |  | 0 | 0 | 0 | 0 | 1 | β | 0 |
| 2 | 69 | f | Non-AD | Non-demented |  | 0 | 0 | 0 | 0 | 0 | NA | 0 |
| 3 | 68 | m | Non-AD (+PART) | Non-demented |  | 1 | 0 | 0 | 0 | 0 | NA | 0 |
| 4 | 74 | m | Non-AD (+infarcts, microinfarcts) | Non-demented |  | 0 | 0 | 0 | 0 | 0 | NA | 0 |
| 5 | 36 | m | Non-AD | Non-demented |  | 0 | 0 | 0 | 0 | 0 | NA | 0 |
| 6 | 69 | f | Non-AD (+PART) | Non-demented |  | 1 | 0 | 0 | 0 | 2 | β | 0 |
| 7 | 59 | f | Non-AD (+PART, CAA) | Non-demented |  | 1 | 0 | 0 | 0 | 0 | NA | 3 |
| 8 | 54 | m | Non-AD (+PART) | Non-demented |  | 1 | 0 | 0 | 0 | 0 | NA | 0 |
| 9 | 56 | m | Non-AD (+PART) | Non-demented |  | 1 | 0 | 0 | 0 | 0 | NA | 0,5 |
| 10 | 76 | m | Non-AD (+PART) | Non-demented |  | 1 | 0 | 0 | 0 | 0 | NA | 1 |
| 11 | 63 | m | Non-AD (+PART) | Non-demented |  | 1 | 0 | 0 | 0 | 0 | NA | na |
| 12 | 58 | f | Non-AD (+PART) | Non-demented |  | 1 | 0 | 0 | 0 | 0 | NA | na |
| 13 | 66 | m | Non-AD (+PART) | Non-demented |  | 1 | 0 | 0 | 0 | 0 | NA | na |
| 14 | 67 | f | Non-AD (+PART) | Non-demented |  | 1 | 0 | 0 | 0 | 0 | NA | na |
| 15 | 56 | f | Non-AD (+PART) | Non-demented |  | 1 | 0 | 0 | 0 | 0 | NA | na |
| 16 | 78 | m | Non-AD (+PART) | Non-demented |  | 2 | 0 | 0 | 0 | 2 | β | na |
| 17 | 53 | f | Non-AD | Non-demented |  | 0 | 0 | 0 | 0 | 0 | NA | na |
| 18 | 55 | f | Non-AD (+PART) | Non-demented |  | 1 | 0 | 0 | 0 | 0 | NA | na |
| 19 | 51 | f | Non-AD (+PART) | Non-demented |  | 1 | 0 | 0 | 0 | 0 | NA | na |
| 20 | 53 | f | Non-AD (+PART) | Non-demented |  | 1 | 0 | 0 | 0 | 0 | NA | na |
| 21 | 78 | f | p-preAD (+CBD) | Non-demented |  | 1 | 1 | 0 | 1 | 2 | β | 3 |
| 22 | 81 | m | p-preAD (+AGD) | Non-demented |  | 2 | 3 | 0 | 1 | 0 | NA | 2 |
| **Case no.** | **Age** | **Sex** | **Neuropathological diagnosis** | **Clinical diagnosis** | **Mutation** | **Braak NFT stage** [1] | **AβMTL phase** [8] | **CERAD score** [5] | **NIA-AA degree** [2] | **LATE-NC stage** [7] | **TDP-43 subtype** [3] | **CDR** [4, 6] |
| 23 | 70 | m | p-preAD | Non-demented |  | 1 | 1 | 0 | 1 | 2 | β | 0 |
| 24 | 73 | f | p-preAD | Non-demented |  | 1 | 1 | 0 | 1 | 1 | β | 0.5 |
| 25 | 83 | f | p-preAD (AGD) | Non-demented |  | 3 | 1 | 0 | 1 | 2 | β | 0 |
| 26 | 72 | m | p-preAD | Non-demented |  | 3 | 2 | 0 | 1 | 1 | β | 0 |
| 27 | 67 | m | p-preAD | Non-demented |  | 2 | 3 | 0 | 1 | 1 | β | 0 |
| 28 | 79 | f | p-preAD (+infarcts) | Non-demented |  | 2 | 0 | 0 | 0 | 2 | β | 2 |
| 29 | 85 | m | p-preAD (+AGD) | Non-demented |  | 3 | 2 | 1 | 1 | 2 | β | 0 |
| 30 | 83 | f | p-preAD | Non-demented |  | 4 | 3 | 1 | 2 | 2 | β | 0 |
| 31 | 77 | m | p-preAD | Non-demented |  | 2 | 1 | 0 | 1 | 2 | β | 0 |
| 32 | 75 | m | p-preAD | Non-demented |  | 1 | 4 | 0 | 1 | 0 | NA | 0 |
| 33 | 73 | m | p-preAD | Non-demented |  | 1 | 1 | 0 | 1 | 0 | NA | 0 |
| 34 | 78 | f | p-preAD (+infarcts, CAA, LBD) | Non-demented |  | 3 | 4 | 1 | 2 | 2 | β | 0 |
| 35 | 75 | f | p-preAD | Non-demented |  | 1 | 2 | 0 | 1 | 0 | NA | 0 |
| 36 | 71 | m | p-preAD | Non-demented |  | 1 | 3 | 0 | 1 | 0 | NA | 0 |
| 37 | 83 | m | AD^TDP-^ (+microinfarcts, CAA) | AD |  | 4 | 4 | 2 | 2 | 0 | NA | 1 |
| 38 | 79 | f | AD^TDP-^ (+microinfarcts) | AD |  | 4 | 3 | 2 | 2 | 0 | NA | na |
| 39 | 87 | f | AD^TDP-^ (+CAA) | AD |  | 4 | 4 | 1 | 2 | 0 | NA | 3 |
| 40 | 72 | f | AD^TDP-^ | AD |  | 4 | 4 | 2 | 2 | 0 | NA | 1 |
| 41 | 78 | f | AD^TDP-^ (+LBD, infarcts) | AD |  | 5 | 4 | 2 | 3 | 0 | NA | 3 |
| 42 | 85 | m | AD^TDP-^ (+CAA, microinfarcts) | AD |  | 3 | 3 | 1 | 2 | 0 | NA | 2 |
| 43 | 81 | m | AD^TDP-^ (+AGD) | AD |  | 3 | 3 | 0 | 2 | 0 | NA | 2 |
| 44 | 83 | m | AD^TDP-^ (+microinfarcts) | AD |  | 2 | 4 | 1 | 1 | 0 | NA | 1 |
| 45 | 82 | m | AD^TDP-^ | AD |  | 2 | 3 | 1 | 1 | 0 | NA | 3 |
| 46 | 54 | f | AD^TDP-^ | AD |  | 6 | 3 | 2 | 3 | 0 | NA | 3 |
| 47 | 68 | f | AD^TDP+CTF^ | AD |  | 6 | 4 | 2 | 3 | 1 | β | 1 |
| **Case no.** | **Age** | **Sex** | **Neuropathological diagnosis** | **Clinical diagnosis** | **Mutation** | **Braak NFT stage** [1] | **AβMTL phase** [8] | **CERAD score** [5] | **NIA-AA degree** [2] | **LATE-NC stage** [7] | **TDP-43 subtype** [3] | **CDR** [4, 6] |
| 48 | 64 | f | AD^TDP+CTF^ | AD |  | 6 | 4 | 3 | 3 | 2 | β | na |
| 49 | 78 | m | AD^TDP+CTF^ (+CAA) | AD |  | 4 | 3 | 1 | 2 | 2 | β | 1 |
| 50 | 98 | f | AD^TDP+CTF^ | AD |  | 4 | 4 | 1 | 2 | 2 | β | 0.5 |
| 51 | 72 | f | AD^TDP+CTF^ | AD |  | 6 | 4 | 3 | 3 | 2 | β | 3 |
| 52 | 82 | m | AD^TDP+CTF^ | AD |  | 3 | 3 | 2 | 2 | 2 | β | 2 |
| 53 | 81 | f | AD^TDP+CTF^ (+CAA) | AD |  | 5 | 4 | 1 | 2 | 2 | β | 3 |
| 54 | 83 | m | AD^TDP+CTF^ | AD |  | 4 | 4 | 3 | 2 | 2 | β | 3 |
| 55 | 87 | m | AD^TDP+CTF^ | AD |  | 6 | 4 | 2 | 3 | 2 | β | 2 |
| 56 | 71 | m | AD^TDP+CTF^ | AD |  | 5 | 4 | 3 | 3 | 2 | β | 3 |
| 57 | 57 | m | AD^TDP+CTF^ | AD |  | 6 | 4 | 3 | 3 | 2 | β | 3 |
| 58 | 55 | f | AD^TDP+CTF^ | AD |  | 6 | 4 | 3 | 3 | 0 | β | 3 |
| 59 | 86 | m | AD^TDP+CTF^ (+infarcts, microinfarcts) | AD+behavioral problems |  | 5 | 4 | 3 | 3 | 2 | β | 2 |
| 60 | 76 | m | AD^TDP+CTF^ | AD |  | 5 | 4 | 3 | 3 | 3 | β | 3 |
| 61 | 77 | f | AD^TDP+CTF^ (+LBD) | AD |  | 5 | 4 | 2 | 3 | 3 | β | 2 |
| 62 | 74 | m | AD^TDP+CTF^ | AD |  | 6 | 4 | 2 | 3 | 3 | β | 2 |
| 63 | 71 | f | AD^TDP+CTF^ | AD |  | 6 | 4 | 2 | 3 | 3 | β | 3 |
| 64 | 76 | m | AD^TDP+/FTLD-^ | AD |  | 5 | 4 | 3 | 3 | 2 | β | 3 |
| 65 | 74 | f | AD^TDP+CTF^ | AD |  | 6 | 4 | 2 | 3 | 2 | β | na |
| 66 | 78 | f | AD^TDP+CTF^ (+CAA) | AD |  | 5 | 4 | 3 | 3 | 3 | β | na |
| 67 | 62 | m | AD^TDP+CTF -^ | AD+motor speech deficits |  | 6 | 4 | 3 | 3 | 2 | β | 1 |
| 68 | 87 | m | AD^TDP+CTF^ | AD |  | 4 | 4 | 2 | 2 | 3 | β | 2 |
| 69 | 64 | m | AD^TDP+CTF^ | AD |  | 6 | 4 | 3 | 3 | 2 | β | 1 |
| 70 | 86 | f | AD^TDP+CTF^ (+AGD) | AD |  | 4 | 4 | 2 | 2 | 2 | β | 2 |
| 71 | 78 | f | AD^TDP+CTF^ | AD |  | 5 | 4 | 2 | 3 | 2 | β | 3 |
| 72 | 89 | f | AD^TDP+CTF^ (+CAA) | AD |  | 4 | 4 | 3 | 2 | 2 | β | 2 |
| **Case no.** | **Age** | **Sex** | **Neuropathological diagnosis** | **Clinical diagnosis** | **Mutation** | **Braak NFT stage** [1] | **AβMTL phase** [8] | **CERAD score** [5] | **NIA-AA degree** [2] | **LATE-NC stage** [7] | **TDP-43 subtype** [3] | **CDR** [4, 6] |
| 73 | 69 | m | AD^TDP+CTF^ | svPPA |  | 6 | 4 | 2 | 3 | 3 | β | 1 |
| 74 | 71 | m | AD^TDP+CTF^ (+LBD) | svPPA |  | 4 | 4 | 3 | 2 | 3 | β | na |
| 75 | 71 | m | AD^TDP+CTF^ (+microinfarcts, CAA) | svPPA |  | 6 | 4 | 2 | 3 | 2 | β | 2 |
| 76 | 83 | f | AD^TDP+CTF^ | svPPA |  | 5 | 4 | 2 | 3 | 3 | β | 3 |
| 77 | 84 | m | AD^TDP+FL^ (+ FTLD-TDP type A) | AD |  | 6 | 4 | 3 | 3 | 3 | α+β | 3 |
| 78 | 68 | m | AD^TDP+FL^ (+ FTLD-TDP type A) | bvFTD |  | 4 | 4 | 2 | 2 | 3 | α+β | 3 |
| 79 | 72 | f | AD^TDP+FL^ (+ FTLD-TDP type C) | bvFTD |  | 3 | 4 | 1 | 2 | 3 | α+β | 3 |
| 80 | 85 | m | AD^TDP+FL^ (+ FTLD-TDP type A) | AD |  | 5 | 4 | 2 | 3 | 2 | α+β | 2 |
| 81 | 75 | M | AD^TDP+FL^ (+ FTLD-TDP type B) | bvFTD+AD | C9ORF72 | 4 | 3 | 1 | 2 | 3 | α+β | 2 |
| 82 | 74 | m | AD^TDP+FL^ (+ FTLD-TDP type A) | AD |  | 6 | 4 | 3 | 3 | 3 | α+β | 3 |
| 83 | 82 | m | AD^TDP+FL^ (+ FTLD-TDP type A) | svPPA+AD |  | 4 | 2 | 2 | 2 | 3 | α+β | 3 |
| 84 | 89 | f | AD^TDP+FL^ (+CAA) | AD |  | 5 | 4 | 3 | 3 | 2 | β | 3 |
| 85 | 83 | m | AD^TDP+FL^ (+CAA) | AD |  | 5 | 4 | 2 | 3 | 2 | β | 3 |
| 86 | 60 | f | AD^TDP+FL^ | AD |  | 5 | 4 | 3 | 3 | 2 | β | 3 |
| 87 | 76 | m | AD^TDP+FL^ | AD |  | 6 | 4 | 3 | 3 | 2 | β | na |
| 88 | 79 | f | FTLD-TDP type A | bvFTD | TBK1 | 1 | 1 | 0 | 1 | NA | NA | 3 |
| 89 | 79 | m | FTLD-TDP type B | AD+behavioral problems |  | 1 | 0 | 0 | 0 | NA | NA | 3 |
| 90 | 62 | f | FTLD-TDP type C | svPPA |  | 0.5 | 0 | 0 | 0 | NA | NA | 3 |
| 91 | 69 | f | FTLD-TDP type C | svPPA |  | 1 | 0 | 0 | 0 | NA | NA | 2 |
| 92 | 64 | f | FTLD-TDP type A | bvFTD | PRG | 1 | 0 | 0 | 0 | NA | NA | 2 |
| 93 | 58 | m | FTLD-TDP type B | bvFTD | C9ORF72 | 1 | 0 | 0 | 0 | NA | NA | 3 |
| 94 | 81 | f | FTLD-TDP type A (+AGD) | svPPA |  | 2 | 2 | 0 | 1 | NA | NA | 2 |
| 95 | 38 | m | FTLD-TDP type A | bvFTD |  | 1 | 0 | 0 | 0 | NA | NA | 2 |
| 96 | 63 | m | FTLD-TDP type A | PSP |  | 2 | 3 | 0 | 1 | NA | NA | 3 |
| 97 | 58 | f | FTLD-TDP type B (+AGD) | bvFTD | C9ORF72 | 1 | 0 | 0 | 1 | NA | NA | 3 |

**Table A2**- List of antibodies used in this study. Abbreviations; IHC: immunohistochemistry, IF: immunofluorescence; Rb: rabbit, Ms: mouse; ND: non-determined, N-t: N-terminal, C-t: C-terminal.

| Antibody name | Epitope | Source (#cat) | Clonality; Clone No. | Host | Dilution | |
| --- | --- | --- | --- | --- | --- | --- |
|  |  |  |  |  | IHC | IF |
| N-t TDP-43 | 1-50aa. | AVIVA systems, San Diego, CA, USA (ARP38941_T100) | Polyclonal | Rb | 1:500 | 1:400 |
| C-t TDP-43 | 260-414aa. | ProteinTech, Rosemont, IL, USA (12892-1-AP) | Polyclonal | Rb | 1:1000 |  |
| C-t TDP-43 | ND | ProteinTech, Rosemont, IL, USA (60019-2-IG) | Monoclonal; 6H6E12 | Ms (IgG1) |  | 1:400 |
| C-t TDP-43 | 405-414aa. | Cosmobiobio Co. LTD, Japan (TIP-PTD-P09) | Polyclonal | Rb | 1:5000 |  |
| pTDP-43^409/410^ | pSer409/pSer410 | Cosmobio Co. LTD, Japan (TIP-PTD-P02) | Polyclonal | Rb | 1:5000 |  |
| pTDP-43^409^ | pSer409 | Cosmobio Co. LTD, Japan (TIP-PTD-P03) | Polyclonal | Rb | 1:5000 |  |
| pTDP-43^403/404^ | pSer403/pSer404 | Cosmobio Co. LTD, Japan (TIP-PTD-P05) | Polyclonal | Rb | 1:500 |  |
| pTDP-43^409/410^ | pSer409/pSer410 | Merck Millipore, Burlington, MA, USA (MABN14) | Monoclonal; 1D3 | Rat | 1:100 |  |
| p-τ | pSer202/pThr205 | Pierce-Endogen | Monoclonal; AT-8 | Mouse | 1/1000 |  |
| p-τ | pSer396/pSer404 | Gift from Dr. Peter Davies | Monoclonal; PHF1 | Ms (IgG1) | 1:500 |  |
| Aβ_17-24_ | 17-24aa. | BioLegend, San Diego, CA, USA (SIG-39220) | Monoclonal; 4G8 | Ms (IgG2b) | 1:5000 |  |

**Table A3**- percentage of positive cases for pTDP-43^409/410^.

| pTDP-43 (S409/S410) | | | | | | |  |  |
| --- | --- | --- | --- | --- | --- | --- | --- | --- |
|  | **non-AD** | **p-preAD** | **AD^TDP-^** | **AD^TDP+CTF^** | **AD^TDP+FL^** | **FTLD-TDP** |  |  |
| **CA1** | 10 | 43,8 | 0 | 90 | 100 | 100 |  | 81-100% |
| **Entorhinal** | 10 | 37,5 | 0 | 76,7 | 100 | 100 |  | 61-80% |
| **Subiculum** | 10 | 31,3 | 0 | 82,8 | 100 | 100 |  | 41-60% |
| **Temporal** | 5 | 25 | 0 | 66,7 | 100 | 100 |  | 21-40% |
| **CA3/2** | 0 | 12,5 | 0 | 66,7 | 100 | 100 |  | 0-20% |
| **NBM** | 0 | 37,5 | 0 | 43,3 | 70 | 100 |  |  |
| **Amygdala** | 5,3 | 6,3 | 0 | 56,7 | 90,9 | 90 |  |  |
| **CA4** | 0 | 6,3 | 0 | 53,3 | 100 | 80 |  |  |
| **DG** | 0 | 6,3 | 0 | 16,7 | 63,6 | 100 |  |  |
| **Frontal** | 0 | 0 | 0 | 33,3 | 50 | 90 |  |  |
| **Occipital** | 0 | 0 | 0 | 14,3 | 77,8 | 77,8 |  |  |

**Table A4**- percentage of positive cases for pTDP-43^409^.

| pTDP-43(S409) | | | | | | |  |  |
| --- | --- | --- | --- | --- | --- | --- | --- | --- |
|  | **non-AD** | **p-preAD** | **AD^TDP-^** | **AD^TDP+CTF^** | **AD^TDP+FL^** | **FTLD-TDP** |  |  |
| **CA1** | 20 | 33,3 | 0 | 90 | 90,9 | 100 |  | 81-100% |
| **Entorhinal** | 10 | 26,7 | 0 | 86,7 | 90,9 | 100 |  | 61-80% |
| **Subiculum** | 10 | 26,7 | 0 | 86,7 | 90,9 | 100 |  | 41-60% |
| **Temporal** | 5 | 20 | 0 | 83,3 | 90,9 | 100 |  | 21-40% |
| **CA3/2** | 0 | 26,7 | 0 | 90 | 90,9 | 100 |  | 0-20% |
| **NBM** | 5 | 0 | 0 | 65,5 | 50 | 60 |  |  |
| **Amygdala** | 0 | 6,3 | 0 | 83,3 | 90,9 | 100 |  |  |
| **CA4** | 0 | 6,7 | 0 | 73,3 | 90,9 | 80 |  |  |
| **DG** | 0 | 6,7 | 0 | 30 | 90,9 | 100 |  |  |
| **Frontal** | 0 | 0 | 0 | 44,8 | 54,5 | 88,9 |  |  |
| **Occipital** | 0 | 0 | 0 | 10 | 45,5 | 55,6 |  |  |

**Table A5**- percentage of positive cases for pTDP-43^403/404^.

| pTDP403/404 | | | | | | |  |  |
| --- | --- | --- | --- | --- | --- | --- | --- | --- |
|  | **non-AD** | **p-preAD** | **AD^TDP-^** | **AD^TDP+CTF^** | **AD^TDP+FL^** | **FTLD-TDP** |  |  |
| **CA1** | 0 | 0 | 0 | 0 | 100 | 100 |  | 81-100% |
| **Entorhinal** | 0 | 0 | 0 | 0 | 100 | 100 |  | 61-80% |
| **Subiculum** | 0 | 0 | 0 | 0 | 100 | 100 |  | 41-60% |
| **Temporal** | 0 | 0 | 0 | 0 | 72,7 | 100 |  | 21-40% |
| **NBM** | 0 | 0 | 0 | 0 | 10 | 50 |  | 0-20% |
| **Amygdala** | 0 | 0 | 0 | 0 | 70 | 100 |  |  |
| **CA4** | 0 | 0 | 0 | 0 | 45,5 | 100 |  |  |
| **DG** | 0 | 0 | 0 | 0 | 45,5 | 66,7 |  |  |
| **CA3/2** | 0 | 0 | 0 | 0 | 54,5 | 100 |  |  |
| **Frontal** | 0 | 0 | 0 | 0 | 63,6 | 90 |  |  |
| **Occipital** | 0 | 0 | 0 | 0 | 18,2 | 60 |  |  |

**Table A6**- percentage of positive cases for C-t-TDP-43.

| C-terminal TDP-43 | | | | | | |  |  |
| --- | --- | --- | --- | --- | --- | --- | --- | --- |
|  | **non-AD** | **p-preAD** | **AD^TDP-^** | **AD^TDP+CTF^** | **AD^TDP+FL^** | **FTLD-TDP** |  |  |
| **CA1** | 0 | 0 | 0 | 3,3 | 90,9 | 100 |  | 81-100% |
| **Entorhinal** | 0 | 0 | 0 | 0 | 72,7 | 100 |  | 61-80% |
| **Subiculum** | 0 | 0 | 0 | 3,3 | 90,9 | 100 |  | 41-60% |
| **Temporal** | 0 | 0 | 0 | 0 | 90,9 | 100 |  | 21-40% |
| **CA3/2** | 0 | 0 | 0 | 0 | 72,7 | 100 |  | 0-20% |
| **NBM** | 0 | 0 | 0 | 0 | 9,1 | 20 |  |  |
| **Amygdala** | 5 | 0 | 0 | 20 | 90,9 | 90 |  |  |
| **CA4** | 0 | 0 | 0 | 0 | 54,5 | 50 |  |  |
| **DG** | 0 | 0 | 0 | 0 | 63,6 | 100 |  |  |
| **Frontal** | 0 | 0 | 0 | 0 | 45,5 | 100 |  |  |
| **Occipital** | 0 | 0 | 0 | 0 | 45,5 | 50 |  |  |

**Table A7**- percentage of positive cases for N-t-TDP-43.

| N-terminal TDP-43 | | | | | | |  |  |
| --- | --- | --- | --- | --- | --- | --- | --- | --- |
|  | **non-AD** | **p-preAD** | **AD^TDP-^** | **AD^TDP+CTF^** | **AD^TDP+FL^** | **FTLD-TDP** |  |  |
| **CA1** | 0 | 0 | 0 | 0 | 81,8 | 100 |  | 81-100% |
| **Entorhinal** | 0 | 0 | 0 | 0 | 45,5 | 100 |  | 61-80% |
| **Subiculum** | 0 | 0 | 0 | 0 | 72,7 | 100 |  | 41-60% |
| **Temporal** | 0 | 0 | 0 | 3 | 63,6 | 100 |  | 21-40% |
| **CA3/2** | 0 | 0 | 0 | 0 | 27,3 | 90 |  | 0-20% |
| **NBM** | 0 | 0 | 0 | 0 | 0 | 30 |  |  |
| **Amygdala** | 0 | 0 | 0 | 2,9 | 70 | 100 |  |  |
| **CA4** | 0 | 0 | 0 | 0 | 18,2 | 80 |  |  |
| **DG** | 0 | 0 | 0 | 0 | 63,6 | 100 |  |  |
| **Frontal** | 0 | 0 | 0 | 0 | 45,5 | 100 |  |  |
| **Occipital** | 0 | 0 | 0 | 0 | 40 | 20 |  |  |

**Table A8**

**a –** Binary logistic regression addressing the differences between AD^TDP+CTF^ vs. AD^TDP+FL^ regarding Braak NFT-staging, when controlled for age and sex.

| **Braak NFT stage** | | | | |
| --- | --- | --- | --- | --- |
|  | **Sig.** | **Odds ratio** | **95%CI for Odds ratio:lower** | **95%CI for Odds ratio:upper** |
| **Braak NFT stage** | 0,397 | 0,698 | 0,303 | 1,6 |
| **Age at death** | 0,917 | 1,004 | 0,921 | 1,097 |
| **Sex** | 0,270 | 0,423 | 0,092 | 1,950 |

**b –** Binary logistic regression addressing the differences between AD^TDP+CTF^ vs. AD^TDP-^regarding Braak NFT-staging, when controlled for age and sex. * after Bonferroni correction for multiple testing.

| **Braak NFT stage** | | | | |
| --- | --- | --- | --- | --- |
|  | **Sig.** | **Odds ratio** | **95%CI for Odds ratio:lower** | **95%CI for Odds ratio:upper** |
| **Braak NFT stage** | 0,005 (0,015)* | 23,761 | 2,61 | 216,3 |
| **Age at death** | 0,026 (0.076)* | 1,206 | 1,022 | 1,423 |
| **Sex** | 0,066 | 19,709 | 0,823 | 472,169 |

**c –** Binary logistic regression addressing the differences between AD^TDP+FL^ vs. AD^TDP-^regarding Braak NFT-staging, when controlled for age and sex.

| **Braak NFT stage** | | | | |
| --- | --- | --- | --- | --- |
|  | **Sig.** | **Odds ratio** | **95%CI for Odds ratio:lower** | **95%CI for Odds ratio:upper** |
| **Braak NFT stage** | 0,050 | 3,165 | 0,999 | 10,031 |
| **Age at death** | 0,940 | 0,995 | 0,880 | 1,126 |
| **Sex** | 0,143 | 5,711 | 0,554 | 58,885 |

**Table A9**

**a –** Binary logistic regression addressing the differences between AD^TDP+CTF^ vs. AD^TDP+FL^ regarding AβMTL phase, when controlled for age and sex.

| **AβMTL phase** | | | | |
| --- | --- | --- | --- | --- |
|  | **Sig.** | **Odds ratio** | **95%CI for Odds ratio:lower** | **95%CI for Odds ratio:upper** |
| **AβMTL phase** | 0,306 | 0,400 | 0,069 | 2,311 |
| **Age at death** | 0,740 | 1,014 | 0,935 | 1,100 |
| **Sex** | 0,423 | 0,524 | 0,408 | 2,544 |

**b –** Binary logistic regression addressing the differences between AD^TDP+CTF^ vs. AD^TDP-^ regarding AβMTL phase, when controlled for age and sex. * after Bonferroni correction for multiple testing.

| **AβMTL phase** | | | | |
| --- | --- | --- | --- | --- |
|  | **Sig.** | **Odds ratio** | **95%CI for Odds ratio:lower** | **95%CI for Odds ratio:upper** |
| **AβMTL phase** | 0,006 (0,018)* | 18,630 | 2,347 | 147,902 |
| **Age at death** | 0,407 | 0,963 | 0,881 | 1,053 |
| **Sex** | 0,362 | 2,344 | 0,376 | 14,607 |

**c –** Binary logistic regression addressing the differences between AD^TDP+FL^ vs. AD^TDP-^ regarding AβMTL phase, when controlled for age and sex.

| **AβMTL phase** | | | | |
| --- | --- | --- | --- | --- |
|  | **Sig.** | **Odds ratio** | **95%CI for Odds ratio:lower** | **95%CI for Odds ratio:upper** |
| **AβMTL phase** | 0,240 | 2,796 | 0,503 | 15,533 |
| **Age at death** | 0,366 | 0,947 | 0,841 | 1,066 |
| **Sex** | 0,149 | 5,085 | 0,560 | 46,189 |

**Table A10**

**a –** Binary logistic regression addressing the differences between AD^TDP+CTF^ vs. AD^TDP+FL^ regarding CERAD score, when controlled for age and sex.

| **CERAD score** | | | | |
| --- | --- | --- | --- | --- |
|  | **Sig.** | **Odds ratio** | **95%CI for Odds ratio:lower** | **95%CI for Odds ratio:upper** |
| **CERAD score** | 0,670 | 0,791 | 0,268 | 2,333 |
| **Age at death** | 0,726 | 1,015 | 0,934 | 1,103 |
| **Sex** | 0,246 | 0,402 | 0,086 | 7,875 |

**b –** Binary logistic regression addressing the differences between AD^TDP+CTF^ vs. AD^TDP-^ regarding CERAD score, when controlled for age and sex. * after Bonferroni correction for multiple testing.

| **CERAD score** | | | | |
| --- | --- | --- | --- | --- |
|  | **Sig.** | **Odds ratio** | **95%CI for Odds ratio:lower** | **95%CI for Odds ratio:upper** |
| **CERAD score** | 0,007 (0,021)* | 12,486 | 2,022 | 77,114 |
| **Age at death** | 0,266 | 1,066 | 0,953 | 1,192 |
| **Sex** | 0,729 | 1,375 | 0,228 | 8,293 |

**c –** Binary logistic regression addressing the differences between AD^TDP+FL^ vs. AD^TDP-^ regarding CERAD score, when controlled for age and sex. * after Bonferroni correction for multiple testing.

| **CERAD score** | | | | |
| --- | --- | --- | --- | --- |
|  | **Sig.** | **Odds ratio** | **95%CI for Odds ratio:lower** | **95%CI for Odds ratio:upper** |
| **CERAD score** | 0,030 (0,087)* | 7,524 | 1,222 | 46,323 |
| **Age at death** | 0,607 | 0,960 | 0,849 | 1,100 |
| **Sex** | 0,118 | 8,990 | 0,573 | 141,170 |

**Table A11**

**a –** Binary logistic regression addressing the differences between AD^TDP+CTF^ vs. AD^TDP+FL^ regarding NIA-AA degree of AD pathology, when controlled for age and sex.

| **NIA-AA degree of AD pathology** | | | | |
| --- | --- | --- | --- | --- |
|  | **Sig.** | **Odds ratio** | **95%CI for Odds ratio:lower** | **95%CI for Odds ratio:upper** |
| **NIA-AA** | 0,850 | 0,859 | 0,179 | 4,115 |
| **Age at death** | 0,686 | 1,017 | 0,936 | 1,107 |
| **Sex** | 0,268 | 0,424 | 0,093 | 1,935 |

**b –** Binary logistic regression addressing the differences between AD^TDP+CTF^ vs. AD^TDP-^ regarding NIA-AA degree of AD pathology, when controlled for age and sex. * after Bonferroni correction for multiple testing.

| **NIA-AA** | | | | |
| --- | --- | --- | --- | --- |
|  | **Sig.** | **Odds ratio** | **95%CI for Odds ratio:lower** | **95%CI for Odds ratio:upper** |
| **NIA-AA** | 0,008 (0,024)* | 33,349 | 2,540 | 437,803 |
| **Age at death** | 0,139 | 1,093 | 0,972 | 1,229 |
| **Sex** | 0,268 | 2,874 | 0,444 | 18,608 |

**c –** Binary logistic regression addressing the differences between AD^TDP+FL^ vs. AD^TDP-^ regarding NIA-AA degree of AD pathology, when controlled for age and sex. * after Bonferroni correction for multiple testing.

| **NIA-AA** | | | | |
| --- | --- | --- | --- | --- |
|  | **Sig.** | **Odds ratio** | **95%CI for Odds ratio:lower** | **95%CI for Odds ratio:upper** |
| **NIA-AA** | 0,035 (0,101)* | 11,493 | 1,193 | 110,700 |
| **Age at death** | 0,741 | 0,980 | 0,867 | 1,107 |
| **Sex** | 0,105 | 8,983 | 0,630 | 128,149 |

**Table A12** – Binary logistic regression addressing the association of the TDP+CTF pattern with Joseph’s TDP-43 types α and β, controlled for age at death and sex. Cases with type α+β were considered as positive for type α as well as for type β, respectively.

| **TDP+CTF Pattern** | | | | |
| --- | --- | --- | --- | --- |
|  | **Sig.** | **Odds ratio** | **95%CI for Odds ratio:lower** | **95%CI for Odds ratio:upper** |
| **TDP-43 Type α** | 0,999 | <0,0001 | <0,0001 | <0,0001 |
| **TDP-43 Type β** | p<0,0001 | 61,326 | 11,509 | 326,789 |
| **Age at death** | 0,185 | 1,049 | 0,977 | 1,127 |
| **Sex** | 0,176 | 3,155 | 0,598 | 16,633 |

**Table A13** – Binary logistic regression addressing the association of the TDP+FL pattern with Josephs’ TDP-43 types α and β, controlled for age at death and sex. Cases with type α+β were considered as positive for type α as well as for type β, respectively.

| **TDP+FL Pattern** | | | | |
| --- | --- | --- | --- | --- |
|  | **Sig.** | **Odds ratio** | **95%CI for Odds ratio:lower** | **95%CI for Odds ratio:upper** |
| **TDP-43 Type α** | 0,999 | >100 | <0,0001 | <0,0001 |
| **TDP-43 Type β** | 0,998 | >100 | <0,0001 | <0,0001 |
| **Age at death** | 0,655 | 1,027 | 0,914 | 1,154 |
| **Sex** | 0,843 | 1,232 | 0,156 | 9,745 |

**Table A14 –** P values of comparisons between TDP-43 antibodies for non-demented cases, n=36. Friedman test with Bonferroni correction for multiple testing. Abbreviations; NBM: basal nucleus of Meynert, DG: dentate gyrus.

| **Non-demented** | | | | | | | |
| --- | --- | --- | --- | --- | --- | --- | --- |
|  | **pTDP409/410 vs pTDP409** | **pTDP409/410 vs pTDP403/404** | **pTDP409/410 vs C-t TDP** | **pTDP409/410 vs N-t TDP** | **pTDP409 vs pTDP403/404** | **pTDP409 vs C-t TDP** | **pTDP409 vs N-t TDP** |
| **CA1** | 1 | 0,890 | 0,890 | 0,890 | 0,890 | 0,890 | 0,890 |
| **Subiculum** | 1 | 1 | 1 | 1 | 1 | 1 | 1 |
| **Entorhinal** | 1 | 1 | 1 | 1 | 1 | 1 | 1 |
| **Temporal** | 1 | 1 | 1 | 1 | 1 | 1 | 1 |
| **CA3/2** | 1 | 1 | 1 | 1 | 1 | 1 | 1 |
| **CA4** | 1 | 1 | 1 | 1 | 1 | 1 | 1 |
| **NBM** | 1 | 1 | 1 | 1 | 1 | 1 | 1 |
| **Frontal** | 1 | 1 | 1 | 1 | 1 | 1 | 1 |
| **Amygdala** | 1 | 1 | 1 | 1 | 1 | 1 | 1 |
| **Occipital** | 1 | 1 | 1 | 1 | 1 | 1 | 1 |
| **DG** | 1 | 1 | 1 | 1 | 1 | 1 | 1 |

**Table A15 –** P values of comparisons between TDP-43 antibodies groups for symptomatic AD cases, n=33. Friedman test with Bonferroni correction for multiple testing. Abbreviations; NBM: basal nucleus of Meynert, DG: dentate gyrus.

| **SympAD** | | | | | | | |
| --- | --- | --- | --- | --- | --- | --- | --- |
|  | **pTDP409/410 vs pTDP409** | **pTDP409/410 vs pTDP403/404** | **pTDP409/410 vs C-t TDP** | **pTDP409/410 vs N-t TDP** | **pTDP409 vs pTDP403/404** | **pTDP409 vs C-t TDP** | **pTDP409 vs N-t TDP** |
| **CA1** | 1 | 0,001 | 0,001 | 0,001 | 0,002 | 0,002 | 0,001 |
| **Subiculum** | 1 | 0,004 | 0,004 | 0,001 | 0,008 | 0,008 | 0,002 |
| **Entorhinal** | 1 | 0,009 | 0,001 | <0,0001 | 0,005 | 0,001 | <0,0001 |
| **Temporal** | 1 | 0,015 | 0,047 | 0,027 | 0,004 | 0,015 | 0,008 |
| **CA3/2** | 1 | 0,005 | 0,009 | 0,002 | <0,0001 | 0,001 | <0,0001 |
| **CA4** | 1 | 0,030 | 0,030 | 0,009 | 0,002 | 0,001 | 0,001 |
| **NBM** | 1 | 0,111 | 0,065 | 0,065 | 0,011 | 0,002 | 0,006 |
| **Frontal** | 1 | 1 | 1 | 1 | 0,753 | 0,297 | 0,297 |
| **Amygdala** | 1 | 0,040 | 1 | 0,127 | <0,0001 | 0,040 | 0,001 |
| **Occipital** | 1 | 1 | 1 | 1 | 1 | 1 | 1 |
| **DG** | 1 | 1 | 1 | 1 | 0,376 | 0,588 | 0,376 |

**Table A16 –** P values of comparisons between TDP-43 antibodies groups for AD/FTD cases, n=5. Friedman test with Bonferroni correction for multiple testing. Abbreviations; NBM: basal nucleus of Meynert, DG: dentate gyrus.

| **AD/FTD** | | | | | | | |
| --- | --- | --- | --- | --- | --- | --- | --- |
|  | **pTDP409/410 vs pTDP409** | **pTDP409/410 vs pTDP403/404** | **pTDP409/410 vs C-t TDP** | **pTDP409/410 vs N-t TDP** | **pTDP409 vs pTDP403/404** | **pTDP409 vs C-t TDP** | **pTDP409 vs N-t TDP** |
| **CA1** | 0,061 | 0,061 | 0,061 | 0,061 | 0,061 | 0,061 | 0,061 |
| **Subiculum** | 0,061 | 0,061 | 0,061 | 0,061 | 0,061 | 0,061 | 0,061 |
| **Entorhinal** | 0,061 | 0,061 | 0,061 | 0,061 | 0,061 | 0,061 | 0,061 |
| **Temporal** | 0,061 | 0,061 | 0,061 | 0,061 | 0,061 | 0,061 | 0,061 |
| **CA3/2** | 0,056 | 0,056 | 0,056 | 0,056 | 0,056 | 0,056 | 0,056 |
| **CA4** | 1 | 1 | 1 | 1 | 1 | 1 | 1 |
| **NBM** | 0,171 | 0,171 | 0,171 | 0,171 | 0,171 | 0,171 | 0,171 |
| **Frontal** | 0,948 | 0,948 | 0,948 | 0,948 | 0,948 | 0,948 | 0,948 |
| **Amygdala** | 0,109 | 0,109 | 0,109 | 0,109 | 0,109 | 0,109 | 0,109 |
| **Occipital** | 0,115 | 0,115 | 0,115 | 0,115 | 0,115 | 0,115 | 0,115 |
| **DG** | 0,406 | 0,406 | 0,406 | 0,406 | 0,406 | 0,406 | 0,406 |

**Table A17 –** P values of comparisons between TDP-43 antibodies groups for FTD cases, n=15. Friedman test with Bonferroni correction for multiple testing. Abbreviations; NBM: basal nucleus of Meynert, DG: dentate gyrus.

| **FTD** | | | | | | | |
| --- | --- | --- | --- | --- | --- | --- | --- |
|  | **pTDP409/410 vs pTDP409** | **pTDP409/410 vs pTDP403/404** | **pTDP409/410 vs C-t TDP** | **pTDP409/410 vs N-t TDP** | **pTDP409 vs pTDP403/404** | **pTDP409 vs C-t TDP** | **pTDP409 vs N-t TDP** |
| **CA1** | 0,064 | 0,064 | 0,064 | 0,064 | 0,064 | 0,064 | 0,064 |
| **Subiculum** | 0,064 | 0,064 | 0,064 | 0,064 | 0,064 | 0,064 | 0,064 |
| **Entorhinal** | 0,064 | 0,064 | 0,064 | 0,064 | 0,064 | 0,064 | 0,064 |
| **Temporal** | 1 | 1 | 1 | 1 | 1 | 1 | 1 |
| **CA3/2** | 1 | 1 | 1 | 0,433 | 1 | 1 | 1 |
| **CA4** | 1 | 1 | 1 | 1 | 1 | 0,730 | 1 |
| **NBM** | 1 | 0,833 | 0,209 | 0,209 | 1,000 | 0,833 | 0,833 |
| **Frontal** | 0,064 | 0,064 | 0,064 | 0,064 | 0,064 | 0,064 | 0,064 |
| **Amygdala** | 0,079 | 0,079 | 0,079 | 0,079 | 0,079 | 0,079 | 0,079 |
| **Occipital** | 1 | 1 | 1 | 1 | 1 | 1 | 1 |
| **DG** | 1 | 1 | 1 | 1 | 1 | 1 | 1 |

**Table A18 –** Multinomial logistic regressions addressing the differences between disease groups, for pTDP^409/410^, in CA1 region. Symptomatic AD cases were considered as the reference category for comparison. Fields were marked with “-” when the software could not assess the statistical value due to overflow.

| **pTDP409/410** | | | | | |
| --- | --- | --- | --- | --- | --- |
| **Symptomic AD vs.** | | **Sig.** | **Odds ratio** | **95%CI for Odds ratio:lower** | **95%CI for Odds ratio:upper** |
| **Non-AD** | **pTDP409/410** | 8,000 | 0,224 | 0,074 | 0,672 |
|  | **Age at death** | 0,016 | 0,933 | 0,881 | 0,987 |
|  | **Sex** | 0,986 | 1,009 | 0,349 | 2,920 |
| **AD/FTD** | **pTDP409/410** | - | - | - | - |
|  | **Age at death** | 0,256 | 0,936 | 0,834 | 1,049 |
|  | **Sex** | - | - | - | - |
| **FTD** | **pTDP409/410** | 0,023 | 16,668 | 1,464 | >100 |
|  | **Age at death** | 0,001 | 0,878 | 0,815 | 0,946 |
|  | **Sex** | 0,964 | 0,969 | 0,249 | 3,764 |

**Table A19 –** Multinomial logistic regressions addressing the differences between disease groups, for pTDP^409^, in CA1 region. Symptomatic AD cases were considered as the reference category for comparison. Fields were marked with “-” when the software could not assess the statistical value due to overflow.

| **pTDP409** | | | | | |
| --- | --- | --- | --- | --- | --- |
| **Symptomic AD vs.** | | **Sig.** | **Odds ratio** | **95%CI for Odds ratio:lower** | **95%CI for Odds ratio:upper** |
| **Non-AD** | **pTDP409** | 0,001 | 0,145 | 0,047 | 0,456 |
|  | **Age at death** | 0,001 | 0,903 | 0,852 | 0,960 |
|  | **Sex** | 0,415 | 0,632 | 0,210 | 1,905 |
| **AD/FTD** | **pTDP409** | - | - | - | - |
|  | **Age at death** | 0,656 | 0,975 | 0,870 | 1,090 |
|  | **Sex** | - | - | - | - |
| **FTD** | **pTDP409** | 0,087 | 6,952 | 0,754 | 64,089 |
|  | **Age at death** | 0,002 | 0,900 | 0,842 | 0,963 |
|  | **Sex** | 0,630 | 1,382 | 0,370 | 5,161 |

**Table A20 –** Multinomial logistic regressions addressing the differences between disease groups, for pTDP^403/404^, in CA1 region. Symptomatic AD cases were considered as the reference category for comparison. Fields were marked with “-” when the software could not assess the statistical value due to overflow.

| **pTDP403/404** | | | | | |
| --- | --- | --- | --- | --- | --- |
| **Symptomic AD vs.** | | **Sig.** | **Odds ratio** | **95%CI for Odds ratio:lower** | **95%CI for Odds ratio:upper** |
| **Non-AD** | **pTDP403/404** | 0,997 | <0,0001 | <0,0001 | <0,0002 |
|  | **Age at death** | 0,001 | 0,906 | 0,857 | 0,958 |
|  | **Sex** | 0,687 | 0,811 | 0,293 | 2,247 |
| **AD/FTD** | **pTDP403/404** | 0,066 | 6,969 | 0,878 | 55,344 |
|  | **Age at death** | 0,470 | 0,961 | 0,861 | 1,071 |
|  | **Sex** | - | - | - | - |
| **FTD** | **pTDP403/404** | 0,001 | 12,648 | 2,719 | 58,833 |
|  | **Age at death** | 0,012 | 0,909 | 0,844 | 0,979 |
|  | **Sex** | 0,893 | 1,109 | 0,246 | 4,999 |

**Table A21 –** Multinomial logistic regressions addressing the differences between disease groups, for C-t TDP-43, in CA1 region. Symptomatic AD cases were considered as the reference category for comparison. Fields were marked with “-” when the software could not assess the statistical value due to overflow.

| **C-t TDP-43** | | | | | |
| --- | --- | --- | --- | --- | --- |
| **Symptomic AD vs.** | | **Sig.** | **Odds ratio** | **95%CI for Odds ratio:lower** | **95%CI for Odds ratio:upper** |
| **Non-AD** | **C-t TDP-43** | 0,997 | <0,0001 | <0,0001 | <0,0001 |
|  | **Age at death** | 0,001 | 0,910 | 0,861 | 0,961 |
|  | **Sex** | 0,631 | 0,780 | 0,283 | 2,150 |
| **AD/FTD** | **C-t TDP-43** | 0,055 | 7,798 | 0,956 | 63,602 |
|  | **Age at death** | 0,395 | 0,953 | 0,852 | 1,065 |
|  | **Sex** | - | - | - | - |
| **FTD** | **C-t TDP-43** | 0,001 | 18,099 | 3,424 | 95,685 |
|  | **Age at death** | 0,004 | 0,894 | 0,828 | 0,965 |
|  | **Sex** | 0,795 | 1,225 | 0,265 | 5,662 |

**Table A22 –** Multinomial logistic regressions addressing the differences between disease groups, for N-t TDP-43, in CA1 region. Symptomatic AD cases were considered as the reference category for comparison. Fields were marked with “-” when the software could not assess the statistical value due to overflow.

| **N-t TDP-43** | | | | | |
| --- | --- | --- | --- | --- | --- |
| **Symptomic AD vs.** | | **Sig.** | **Odds ratio** | **95%CI for Odds ratio:lower** | **95%CI for Odds ratio:upper** |
| **Non-AD** | **N-t TDP-43** | 0,997 | <0,0001 | <0,0001 | <0,0001 |
|  | **Age at death** | 0,001 | 0,905 | 0,856 | 0,957 |
|  | **Sex** | 0,696 | 0,816 | 0,294 | 2,263 |
| **AD/FTD** | **N-t TDP-43** | 0,203 | 3,976 | 0,474 | 33,372 |
|  | **Age at death** | 0,574 | 0,969 | 0,868 | 1,082 |
|  | **Sex** | - | - | - | - |
| **FTD** | **N-t TDP-43** | 0,001 | 13,321 | 2,874 | 61,747 |
|  | **Age at death** | 0,020 | 0,916 | 0,850 | 0,986 |
|  | **Sex** | 0,945 | 1,055 | 0,231 | 4,815 |

**Table A23** Binary logistic regression addressing the association of Josephs’ TDP-43 pathology type α with FTD symptoms, controlled for age at death and sex. Cases with type α+β were considered as positive for type α as well as for type β, respectively.

| **TDP-43 Type α** | | | | |
| --- | --- | --- | --- | --- |
|  | **Sig.** | **Odds ratio** | **95%CI for Odds ratio:lower** | **95%CI for Odds ratio:upper** |
| **FTD symptoms** | 0,039 | 10,08 | 1,122 | 90,553 |
| **Age at death** | 0,157 | 1,091 | 0,967 | 1,230 |
| **Sex** | 0,102 | 7,074 | 0,677 | 73,913 |

**Table A24** Binary logistic regression addressing the association of Josephs’ TDP-43 pathology type β with FTD symptoms, controlled for age at death and sex. Fields were marked with “-” when the software could not assess the statistical value due to overflow. Cases with type α+β were considered as positive for type α as well as for type β, respectively.

| **TDP-43 Type β** | | | | |
| --- | --- | --- | --- | --- |
|  | **Sig.** | **Odds ratio** | **95%CI for Odds ratio:lower** | **95%CI for Odds ratio:upper** |
| **FTD symptoms** | 0,999 | >100 | <0,0001 | - |
| **Age at death** | 0,005 | 1,071 | 1,020 | 1,124 |
| **Sex** | 0,321 | 1,660 | 0,610 | 4,513 |

**Table A25** Binary logistic regression addressing the association of FTD symptoms with TDP+CTF pattern, controlled for age at death and sex. Only demented cases were considered.

| **FTD symptoms** | | | | |
| --- | --- | --- | --- | --- |
|  | **Sig.** | **Odds ratio** | **95%CI for Odds ratio:lower** | **95%CI for Odds ratio:upper** |
| **TDP+CTF Pattern** | 0,022 | 0,178 | 0,041 | 0,778 |
| **Age at death** | 0,036 | 0,926 | 0,861 | 0,995 |
| **Sex** | 0,225 | 2,476 | 0,573 | 10,702 |

**Table A26 –** Binary logistic regression addressing the association of typical AD symptoms with TDP+FL pattern, controlled for age at death and sex. Only demented cases were considered.

| **Typical AD symptoms** | | | | |
| --- | --- | --- | --- | --- |
|  | **Sig.** | **Odds ratio** | **95%CI for Odds ratio:lower** | **95%CI for Odds ratio:upper** |
| **TDP+FL Pattern** | 0,003 | 0,126 | 0,032 | 0,500 |
| **Age at death** | 0,034 | 1,085 | 1,006 | 1,169 |
| **Sex** | 0,530 | 1,559 | 0,389 | 6,238 |

**References**

1. Braak H, Alafuzov I, Arzberger T, Kretzschmar H, Del Tredici K (2006) Staging of Alzheimer disease-associated neurofibrillary pathology using paraffin sections and immunocytochemistry. Acta Neuropathol 112:389–404. doi: 10.1007/s00401-006-0127-z

2. Hyman BT, Phelps CH, Beach TG, Bigio EH, Cairns NJ, Carrillo MC, Dickson DW, Duyckaerts C, Frosch MP, Masliah E, Mirra SS, Nelson PT, Schneider JA, Thal DR, Thies B, Trojanowski JQ, Vinters H V, Montine TJ (2012) National Institute on Aging-Alzheimer’s Association guidelines for the neuropathologic assessment of Alzheimer’s disease. Alzheimers Dement 8:1–13. doi: 10.1016/j.jalz.2011.10.007

3. Josephs KA, Murray ME, Tosakulwong N, Weigand SD, Serie AM, Ralph PB, Perkerson B, Matchett BJ, Jack Jr CR, David KS, Petersen RC, Parisi JE, Petrucelli L, Baker M, Rademakers R, Whitwell JL, Dickson DW (2019) Pathological, imaging and genetic characteristics support the existence of distinct TDP-43 types in non-FTLD brains. Acta Neuropathol 137:227–238. doi: 10.1007/s00401-018-1951-7

4. Mioshi E, Flanagan E, Knopman D (2017) Detecting clinical change with the CDR-FTLD: differences between FTLD and AD dementia. Int J Geriatr Psychiatry 32:977–982. doi: 10.1002/gps.4556

5. Mirra SS, Heyman A, McKeel D, Sumi SM, Crain BJ, Brownlee LM, Vogel FS, Hughes JP, van Belle G, Berg L, Ball MJ, Bierer LM, Claasen D, Hansen LR, Hart M, Hedreen J, Baltimore B, Hen Derson V, Hyman BT, Joachim C, Mark-Esbery W, Mar Tinez AJ, McKee A, Miller C, Moossy J, Nochlin D, Perl D, Petito C, Rao GR, Schelper RL, Slager U, Terry RD (1991) The consortium to establish a registry for Alzheimer’s disease (CERAD). Part II. Standardization of the neuropathologic assessment of Alzheimer’s disease. Neurology 41:479–486. doi: 10.1212/wnl.41.4.479

6. Morris JC (1993) The Clinical Dementia Rating (CDR): current version and scoring rules. Neurology 43:2412–4. doi: 10.1212/WNL.43.11.2412-A

7. Nelson PT, Dickson DW, Trojanowski JQ, Jack CR, Boyle PA, Arfanakis K, Rademakers R, Alafuzoff I, Attems J, Brayne C, Coyle-Gilchrist ITS, Chui HC, Fardo DW, Flanagan ME, Halliday G, Hokkanen SRK, Hunter S, Jicha GA, Katsumata Y, Kawas CH, Keene CD, Kovacs GG, Kukull WA, Levey AI, Makkinejad N, Montine TJ, Murayama S, Murray ME, Nag S, Rissman RA, Seeley WW, Sperling RA, White Iii CL, Yu L, Schneider JA (2019) Limbic-predominant age-related TDP-43 encephalopathy (LATE): consensus working group report. Brain 0:1–25. doi: 10.1093/brain/awz099

8. Thal DR, Schultz C, Sassin I, Ghebremedhin E, Del Tredici K, Braak E, Braak H (2000) Sequence of Aβ-Protein Deposition in the Human Medial Temporal Lobe. J Neuropathol Exp Neurol 59:733–748. doi: 10.1093/jnen/59.8.733
